# Combating hypertension beyond genome-wide association studies: Microbiome and artificial intelligence as opportunities for precision medicine

**DOI:** 10.1017/pcm.2023.13

**Published:** 2023-05-16

**Authors:** Sachin Aryal, Ishan Manandhar, Xue Mei, Beng S. Yeoh, Ramakumar Tummala, Piu Saha, Islam Osman, Jasenka Zubcevic, David J. Durgan, Matam Vijay-Kumar, Bina Joe

**Affiliations:** 1 Center for Hypertension and Precision Medicine, Department of Physiology and Pharmacology, University of Toledo College of Medicine and Life Sciences, Toledo, OH, USA; 2 Integrative Physiology & Anesthesiology, Baylor College of Medicine, Houston, TX, USA

**Keywords:** Machine learning, high blood pressure, gut microbiota, genome-based risk scores, personalized medicine

## Abstract

The single largest contributor to human mortality is cardiovascular disease, the top risk factor for which is hypertension (HTN). The last two decades have placed much emphasis on the identification of genetic factors contributing to HTN. As a result, over 1,500 genetic alleles have been associated with human HTN. Mapping studies using genetic models of HTN have yielded hundreds of blood pressure (BP) loci but their individual effects on BP are minor, which limits opportunities to target them in the clinic. The value of collecting genome-wide association data is evident in ongoing research, which is beginning to utilize these data at individual-level genetic disparities combined with artificial intelligence (AI) strategies to develop a polygenic risk score (PRS) for the prediction of HTN. However, PRS alone may or may not be sufficient to account for the incidence and progression of HTN because genetics is responsible for <30% of the risk factors influencing the etiology of HTN pathogenesis. Therefore, integrating data from other nongenetic factors influencing BP regulation will be important to enhance the power of PRS. One such factor is the composition of gut microbiota, which constitute a more recently discovered important contributor to HTN. Studies to-date have clearly demonstrated that the transition from normal BP homeostasis to a state of elevated BP is linked to compositional changes in gut microbiota and its interaction with the host. Here, we first document evidence from studies on gut dysbiosis in animal models and patients with HTN followed by a discussion on the prospects of using microbiota data to develop a metagenomic risk score (MRS) for HTN to be combined with PRS and a clinical risk score (CRS). Finally, we propose that integrating AI to learn from the combined PRS, MRS and CRS may further enhance predictive power for the susceptibility and progression of HTN.

## Graphical abstract

**Figure:**
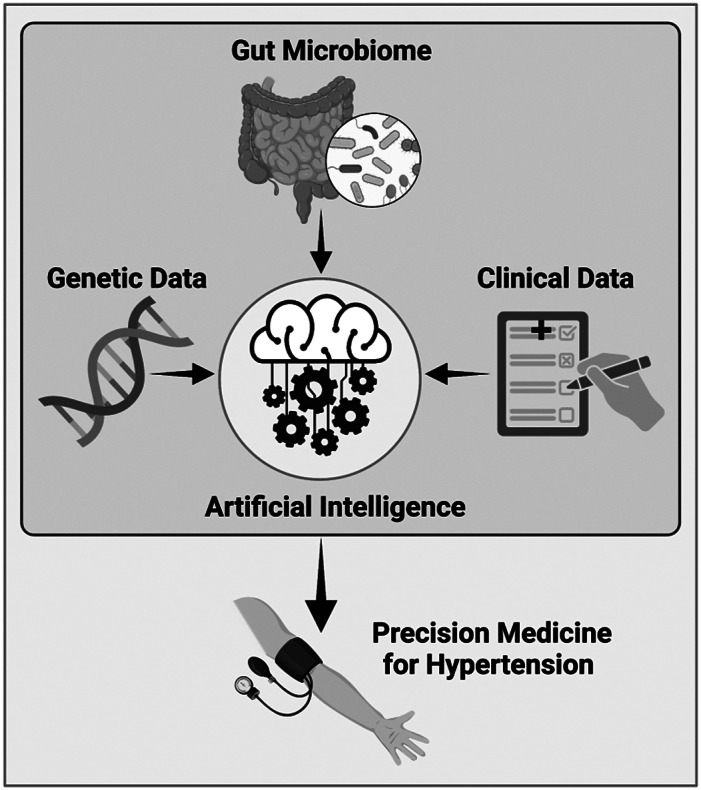

## Impact statement

More than half of the world’s adult population suffers from hypertension (HTN), which is the single largest risk factor for human mortality. Despite available medications, susceptibility to develop HTN has not decreased because current knowledge on the risk assessment for susceptibility is severely limited. In this context, genome-wide association studies for HTN are factoring the genetic contributions toward the development of a polygenic risk score (PRS) for HTN. However, given that nongenetic factors also contribute to the etiology of HTN, PRS alone may be insufficient to account for the incidence and progression of HTN. One such nongenetic factor is gut microbiota, which is acquired at birth and demonstrated to be a definitive link to the etiology of HTN. Therefore, here we discuss the prospects for developing and integrating a microbiota-based ‘metagenomic risk score’ with PRS, and a clinical risk score to construct an artificial intelligence-based model for precision diagnosis and management of HTN.

## Introduction

Despite improvements in health care, cardiovascular disease (CVD) remains the leading cause of human mortality globally (Vos et al., 2020). The propensity to develop CVDs is fueled by chronically elevated blood pressure (BP) or hypertension (HTN). Among others, essential HTN is the most frequent type of HTN in adults (accounts for 95%). It is caused when there is sustained increase in the BP greater than 140/90 mmHg and when no etiology can be determined for the HTN (Gupta-Malhotra et al., 2015). According to the World Health Organization, an estimated 1.28 billion adults of the age-group 30–79 years worldwide suffer from HTN (https://www.who.int/news-room/fact-sheets/detail/hypertension). Therefore, controlling the incidence of HTN is critical for improving the quality of life and prevention of premature death.

Research on HTN over the last few decades has established that the susceptibility to HTN is determined both by genetic and environmental factors. The estimated contribution of heritability of HTN is ~30%, while environmental factors contribute to ~70% (Biino et al., 2013). Despite the relatively lower contributions of genetics to HTN, there has been considerable focus on mining the genomic contributions to the genesis of HTN. There are two major factors propelling the momentum for understanding the genetics of HTN, (i) the desire to find novel druggable targets and (ii) advances in whole-genome sequencing, which alleviated the technical limitation of detecting human genetic variation on a large scale. Such efforts have thus far identified over 1,500 loci in human HTN (Evangelou et al., 2018; Buniello et al., 2019; Cabrera et al., 2019; Giri et al., 2019). However, while they collectively define the genomic landscape for association with HTN in humans, individually, they are not druggable targets because each of these loci contribute very little to BP regulation.

In experimental studies using animal models, the genomic landscape for association with HTN was similar to that of humans. Animal model studies identified over 400 BP quantitative trait loci (https://rgd.mcw.edu/rgdweb/elasticResults.html?term=blood+pressure&chr=ALL&start=&stop=&species=Rat&category=QTL&objectSearch=true). Details on these investigations are documented in our previous review (Padmanabhan and Joe, 2017) and updated in recent articles (Warren et al., 2017; Evangelou et al., 2018; Giri et al., 2019; Surendran et al., 2020; Olczak et al., 2021; Padmanabhan and Dominiczak, 2021). Meanwhile, research beyond genomic analyses has led to the profound realization that gut microbiota is an important nongenomic factor which was not previously accounted for in the etiology of HTN. Specifically, our group was the first to report the evidence of gut microbiota dysbiosis in both hypertensive animal models and patients (Mell et al., 2015; Yang et al., 2015). Following this pioneering discovery, associations between gut microbiota are reported between hypertensive and normotensive animal models and humans (Tables 1 and 2). In this article, we review the literature on gut microbiota and HTN and propose developing a gut metagenomic risk score (MRS) for HTN. Further, we discuss the value of combining MRS with polygenic risk score (PRS), CRS and artificial intelligence (AI) for clinical management of HTN (Graphical Abstract).

**Table 1.** The association observed between animal hypertension, gut microbiota and various interventions

References	Model	Intervention	Effect on blood pressure	Enriched/decreased in HTN	Enriched/decreased in normotensive or treatment group
Yang et al., 2015	SHR versus WKY	Genetic	BP increased in SHR	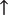 Lactate-producers (*Streptococcus, Turicibacter*) and others *Oribacterium, Parabacteroides*	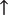 Butyrate producers (*Coprococcus*, *Pseudobutyrivibrio*) and others (*Allobaculum*, *Bifidobacterium*, *Alistipes, Blautia and Bacteroidetes*)
	SD rats	1. Angiotensin II (Ang II) 2. Ang II + Minocycline	Minocycline attenuated Ang II-induced HTN	*Ang II group:* 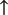 F/B ratio, Firmicutes	*Ang II ± Minocycline:* 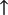 Acetate and butyrate producers; *Akkermansia, Bacteroides, Enterorhabdus* and *Marvinbryantia* 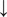 Bacteroidetes
Durgan et al., 2016	Long Evans Rats	High fat diet (HFD) and Obstructive sleep apnea (OSA)	HFD + OSA increased BP	*HFD ± OSA:* 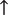 Coriobacteriaceae, *Lactococcus* 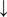 Ruminococcaceae, Clostridaiales	NA
		Cecal content transplant from HTN OSA rats on HFD to normotensive OSA on chow	Microbiotal transplant developed HTN in normotensive OSA on chow	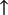 in Coriobacteriaceae 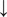 in Eubacterium	NA
Mell et al., 2015	Dahl salt-sensitive (DSS) and salt-resistant (DSR) rats	High salt diet (HSD)	BP was elevated on HSD	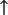 Bacteroidetes, family S24–7, Lactobacillaceae, Veillonellaceae 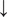 Enterobacteriaceae	NA
		Cecal content transplant from DSR to DSS versus DSS to DSS transfer	Microbiotal transplant from DSR elevated SBP in DSS rats	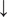 Clostridiales, Veillonellaceae, Mollicutes	NA
Santisteban et al., 2017	Young pre-hypertensive SHR versus young WKY	N/A	Young pre-hypertensive SHR	No significant changes in gut microbiota	NA
Santisteban et al., 2017	Adult SHR versus WKY	N/A	BP elevated in SHR	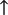 Parabacteroides, Porphyromonadaceae, Lactobacillaceae, *Streptococcus*, Streptococcaceae, Latobacillales, *Mogibacterium*, *Oribacterium*, *Turibacter*	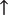 Bifidobacterium, Bifidobacteriaceae, Bifidobacteriales, *Gordonibacter*, Bacteroides, Bacteroidaceae, Prevotellaceae, *Alistipes*, Rikenellaceae, Bacteroidales, *Anaerotruncus*, *Dorea*, *Blautia*, *Coprococcus*, Lachnospira, *Allobaculum*, *Coprabacillus, Massilia*, Oxalobacteraceae, *Escherichia_Shigella*, Enterobacteriaceae, Enterobacteriales
Adnan et al., 2017	WKY rats	FMT from SHRSP	SBP increased by FMT	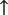 Erysipelotrichaceae, Dorea, Anaerostipes, Bacteroidales, Micrococcaceae, *Ruminococcus*, Deferribacterales, Deferribacteres, *Mucispirillum*, Deferribacteraceae, Deferribacteres, *Lactococcus*, *Desulfovibrio*, Deltaproteobacteria, Desulfovibrionales, *Roseburia*, *Coprococcus*, Lachnospiraceae, Clostridiales, Firmicutes 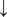 Bacteroidetes, Bacteroidia, Erysipelotrichi, Erysipelotrichales, *Allobaculum*, Actinobacteria, Bacteroidaceae, Bacteroides, Actinobacteria, Bifidobacteriales, *Bifidobacterium*, Enterobacteriales, Gammaproteobacteria, Enterobacteriaceae, Betaproteobacteria, *Sutterella*, Alcaligenaceae, Bacillales, Bacillaceae, *Coprobacillus*, Coriobacteriales, Coriobacteriia, *Adlercreutzia*, *Holdemania*, *Enterococcus*	NA
Marques et al., 2017	C57BI/6 mice	High-fiber diet and acetate supplementation in Deoxycorticosterone acetate (DOCA) model	SBP and DBP reduced by high-fiber and acetate supplementation in DOCA-induced HTN	*DOCA-control:* 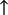 YS2	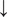 F/B ratio 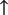 *Bacteroides acidifaciens*, acetate-producing bacteria
Sherman et al., 2018	Wistar rats	Prenatal androgen (PNA) exposure in induced hypertension in offspring	Increased BP in PNA-exposed rats	*Actinobacteria:* 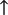 Yaniellaceae, Geodermatophilaceae, Microbacteriaceae, Nakamurellaceae, Corynebacteriaceae, Promicromonosporaceae and Nocardiaceae 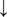 Brevibacteriaceae and Dermabacteraceae *Bacteroidetes:* 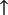 Rikenellaceae, Paraprevotellaceae 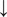 Bacteroidaceae, Odoribacteraceae and *S24–7* were significantly decreased *Firmicutes:* 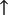 Peptococcaceae, Eubacteriaceae, Carnobacteriaceae, Tissierellaceae, Streptococcaceae, Veillonellaceae, Coprobacillus and Leuconostocaceae 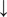 Ruminococcaceae, Lachnospiraceae, Clostridiaceae, Erysipelotrichaceae, Dehalobacteriaceae, Lactobacillaceae and Mogibacteriaceae *Verrucomicrobia:* 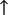 *Verrucomicrobiaceae*	NA
Wilck et al., 2017	FVB/N mice	High salt diet (HSD)	HSD induced HTN	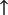 *Parasutterella* spp., *Akkermansia* and *Alistipes* 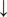 *Lactobacillus,* *Oscillibacter, Pseudoflavonifractor, Clostridium XIVa, Johnsonella and Rothia*	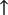 Christensenellaceae, Corynebacteriaceae, *Erwinia*, *Corynebacterium*
Robles-Vera et al., 2018	Wistar rats	NG -Nitro-l-Arginine Methyl Ester (l-NAME)	l-NAME treatment caused progressive increase in SBP and DBP	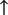 F/B ratio, *Propionibacterium* 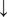 Parabacteroides, *Bifidobacterium*, *Olivibacter*, *Dysgonomonas*, *Pedobacter*, *Flavobacterium* and *Desulfotomaculum*	NA
Toral et al., 2018	C57BI/6 J Mice	Treatment with probiotic *Lactobacillus fermentum* CECT5716 (LC40) in tacrolimus-induced hypertension	Tacrolimus increased SBP and alleviated by LC40 administration	*Tacrolimus group:* 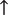 F/B ratio 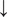 Butyrate- (including *Butyricimonas*) and acetate-producing bacteria in tacrolimus group, *Bifidobacterium*	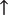 *Bifidobacterium*, (*L. fermentum* CECT5716) 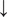 *Anaerostipes*, *Hespellia*, *Prevotella*
Bier et al., 2018	DSS	HSD	BP levels increased in HSD group	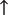 Christensenellaceae, Corynebacteriaceae, *Erwinia*, *Corynebacterium*	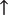 *Anaerostipes*
Waghulde et al., 2018	DSS	RNA CRISPR (Gper1 deletion) Gper1^−/−^	*Gper1* ^−/−^ rats showed significantly lower BP effect	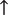 unclassified Clostridiales	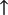 Parabacteroides, Bacteroidales S24–7, unclassified enterobacteriaceae 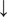 F/B ratio
Galla et al., 2018	DSS	Neomycin	Neomycin elevated BP	*DSS (neomycin):* 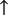 Bacteroidetes, Cyanobacteria, Fusobacteria and Verrucomicrobia 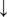 in Actinobacteria, Deferribacteres, Firmicutes, Proteobacteria, TM7 and Tenericutes	NA
	DSS	Minocycline	Minocycline elevated BP	*DSS (minocycline):* 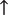 Firmicutes, Proteobacteria and Verrucomicrobia 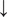 Actinobacteria, Bacteroidetes, Cyanobacteria, Deferribacteres, TM7 and Tenericutes	NA
	DSS	Vancomycine	Vancomycine elevated BP	*DSS (vancomycine):* 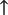 Bacteroidetes, Cyanobacteria, Elusimicrobia, Fusobacteria, Proteobacteria and Verrucomicrobia 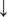 Actinobacteria, Deferribacteres, Firmicutes, TM7 and Tenericutes	NA
	SHR	Neomycin	No change	*SHR (neomycine):* 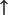 Bacteroidetes, Cyanobacteria, Elusimicrobia and Verrucomicrobia 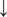 Firmicutes, Proteobacteria, TM7 and Tenericutes	NA
	SHR	Minocycline	Reduced BP	*SHR (minocycline):* 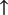 Actinobacteria, Cyanobacteria, Deferribacteres and Firmicutes 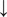 Bacteroidetes, Proteobacteria, TM7 and Tenericutes	NA
	SHR	Vancomycine	Reduced BP	*SHR (vancomycine):* 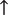 Bacteroidetes, Cyanobacteria, Proteobacteria and Verrucomicrobia 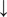 Firmicutes, TM7, and Tenericutes	
Tain et al., 2018	SD rats	Resveratrol treatment in maternal and post-weaning high fat-induced (HF/HF) HTN	Resveratrol attenuated HF/HF-induced HTN	*HF/HF versus control:* 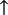 F/B ratio, Verrucomicrobia, *Tepidibacter*, *Lactococcus, Serratia, Enterobacter, Erwinia, Mucispirillum, Akkermansia municiniphila* 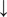 Bacteroidetes, *Turicibacter*, *Lactobacillus*, *Leuconostoc*	*HF/HF ± Resveratrol:* 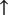 *Flavobacterium, Tepidibacter, Lactococcus* and *Erysipelothrixas* 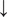 *Acholeplasma* and *Turicibacter*
Sharma et al., 2019	SD rats	Tetracycline (CMT-3) treatment in Angiotensin II HTN	CMT-3 attenuated Ang II-induced HTN	*Ang II versus control:* 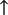 Proteobacteria, Parabacteroides, *Blautia* 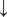 *Ruminococcus*	CMT-3 treatment restored altered taxa in Ang II-induced HTN group
Toral et al., 2019b	SHR and WKY	FTM From WKY to SHR	Reduced basal SBP in SHR after FTM from WKY	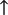 Firmicutes 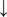 Bacteroidetes, F/B ratio	NA
Hsu et al., 2019	SD rats	Maternal and post-weaning high-fat diet (HF/HF)	HF/HF diet elevated BP	*HF/HF:* 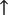 F/B ratio, Verrucomicrobia, *Akkermansia, Clostridium, Alkaliphilus* 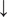 *Lactobacillus*, Parabacteroides, *Ruminococcus*	NA
Yang et al., 2019a	SHR and WKY	Captopril (CAP)	CAP decreased BP in SHR	NA	*SHR ± CAP:* 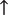 Firmicutes, Proteobacteria, Actinobacteria and Tenericutes, *Mucispirillum*, Parabacteroides, *Allobaculum*
Toral et al., 2019a	SHR and WKY	FTM from SHR to WKY (S-W)	Increased basal SBP and DBP	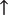 Odoribacteraceae, *Odoribacter*	NA
		FTM from WKY to SHR (W-S)	BP was reduced	NA	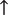 *Blautia*, Peptococcaceae, Lactobacillaceae, *Lactobacillus*, Firmicutes
Yan et al., 2020	Wister rats	HSD	SBP and DBP are significantly higher in HSD group	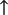 F/B ratio, Spirochates 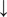 Verrucomicrobia, *Bacteroides fragilis*	N/A
Chen et al., 2020	Sprague–Dawley rats	20% fructose and 4% Nacl (HFS)	Chronic HFS elevated BP	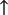 Rikenellaceae, Bacteroidetes 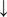 F/B ratio, Desulfovibrionaceae	NA
Xia et al., 2021	SHR	Exercise	Exercise significantly decreased SBP in SHR resembling antihypertensive effects	*SHR-exercise versus SHR-sedentary:* 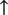 Acetate and Butyrate producers 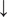 F/B ratio	NA
Robles-Vera et al., 2020a	Wister rats	Effect of 1. Bifidobacterium (BFM) 2. Butyrate 3. Acetate in DOCA-salt-induced HTN	DOCA-salt group with BFM treatment prevented the rise in SBP	*DOCA-salt versus control* **:** 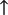 in Actinobacteria, *Blautia*, Peptostreptococcaceae, acetate, butyrate and lactate producers 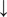 in Rikinellaceae	*DOCA-salt-BFM group:* 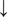 Peptostreptococcaceae resembling the level to that of control group *DOCA-salt-Acetate:* 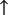 Bacteroides 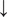 *Blautia, Prevotella*
Li et al., 2020	SHR	Maternal captopril effect in SHR offspring	Maternal treatment with captopril significantly lowered BP in SHR Male offspring compared to SHR control.	*SHR:* 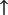 F/B ratio	*In offspring with maternal route ± sustained CAP:* 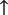 Allobaculum, Erysipelotrichaceae and Erysipelotrichia *In offspring with maternal route only:* 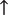 Clostridiales, *Anaerostipes, Coprococcus, Oscillospira, Roseburia, Dehalobacterium.* *SHR offspring with only maternal route of CAP:* 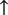 butyrate-producing bacteria, *Coprococcus, Roseburia* and *Oscillospira*
Robles-Vera et al., 2020	WKY	Effect of mycophenolate (MMF) in DOCA-salt-induced HTN	The immunosuppressive drug mofetil MMF significantly reduced BP	*DOCA:* 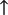 *Bacilli,* Lactobacillaceae, Burkhholderiales, Betaproteobacteria, Alcaligenaceae	*DOCA ± MMF* 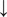 F/B ratio
Hsu et al., 2020	SHR	Effect of maternal *N*-acetylcysteine (NAC) treatment in offspring	Maternal NAC treatment inhibited the rise in SBP	*SHR controls**:*** 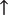 *Bifidobacterium*, *Lactobacillus, Turicibacter, Akkermansia* 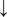 *Holdemania* compared to WKY	*SHR ± NAC:* 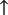 Actinobacteria, *Bifidobacterium* and *Allobaculum* 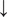 Verrucomicrobia, *Turicibacter,* and *Akkermansia*
Robles-Vera et al., 2020b	SHR and WKY	SHR treated with losartan (Angiotensin receptor antagonist)	Losartan (Los) treated SHR showed progressive reduction in SBP	*SHR group versus WKY* 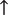 *Lactobacillaceae*, *Lactobacillus,* and other lactate‐producing bacteria 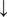 *Verrucomicrobiaceae,* *Pedobacter*, *Akkermansia* and other acetate and propionate‐producing bacteria	*SHR ± Los versus SHR* 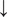 F/B ratio, Verrucomicrobia, 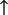 Bacteroidetes, Lactobacillaceae and *Lactobacillus*
Galla et al., 2020	DSS	Amoxicillin administered to young rats	Amoxicillin administration reduced BP	NA	*Amoxicillin versus controls* **:** 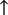 Firmicutes, TM7, Tenericutes, Bacteroidia, Beta Proteobacteria; order: Bacteroidales, Enterobacteriales, and Burkholderiales, Prevotellaceae, Enterococcaceae, Enterobacteriaceae, Alcaligenaceae, Bacteroidaceae, *Blautia*, *Prevotella*, *Enterobacter*, *Enterococcus*, *Klebsiella*, *Sutterella* and *Bacteroides* 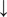 in Bacteroidetes, TM7–3, Clostridia, Delta Proteobacteria, Bacilli, Erysipelotrichia, Gamma Proteobacteria and Mollicutes; order: Cw040, Clostridiales, Desulfovibrionales, Lactobacillales, Erysipelotrichales, Pseudomonadales, Turicibacterales and Bacteroidales, F16, Rumincoccaceae, Clostridiales‐F, Desulphovibrionaceae, Veillonellaceae, Lacnosphiraceae, Mogibacteriaceae, Lactobacillaceae, Erysipelotrichaceae, Pseudomonadaceae, Clostridiaceae, Turicibacteraceae and Peptostreptococcaceae, *Ruminococcus*, *Oscillospira*, *Anaerovibrio*, (*Ruminococcus*), *Lactobacillus*, *Pseudomonas*, *Coprococcus*, *Dorea*, *Clostridium*, *Roseburia*, *Turicibacter* and (*Prevotella*) (from Paraprevotellaceae family).
		Amoxicillin was administered dams (Gestation and lactation)	Amoxicillin administration reduced BP		Significantly 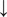 in the amoxicillin‐treated group These include; classes Delta Proteobacteria and TM7_3; orders Cw040 and Bacteriodales; families Veillonellaceae, F16, Desulfovibrionellaceae and Porphyromonadaceae and genus *Parabacteroide* When compared with their maternal microbiota, amoxicillin consistently 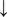 a few groups of bacteria in offspring. F16, Cw040, TM7_3, Veillonellaceae, and Bacteriodales remained consistently reduced in offspring
Chakraborty et al., 2020b	DSS	High salt and day/night effect	BP was high during active (night) phase in both high and low salt groups. High SBP in high salt compared to low salt	*High salt (Dark vs. light):* 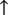 Sutterella, 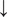 Clostridiales *Low salt (Dark vs. light):* 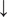 Streptococcaceae and *Lactobacillus* *High salt dark versus low salt dark:* 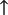 *Sutterella,* Erysipelotrichaceae, Ruminococcaceae, 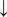 Clostridiales *High salt light versus low salt light:* 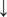 *Lactobacillus* 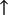 Ruminococcaceae	NA
Abboud et al., 2021	SHR	Cross-fostered SHR by WKY dams	BP was reduced	*Cross-fostered SHR* 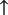 *Escherichia-Shigella* 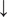 *Haemophilus, Lactobacillus intestinalis, Romboustia, Rothia*	NA
Shi et al., 2021b	SHRSP and WKY	Every other day fasting (EODF)	EODF attenuated BP rise in SHRSP	*SHRSP Control versus SHRSP EODF*: *SHRSP Control*: 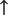 *Bacteroides uniformis*, *Lactobacillus johnsonii*, *Lactobacillus reuteri*, Lachnospiraceae bacterium A4, Oscilibacter sp. *SHRSP EODF*: 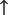 *Asaccharobacter celatus*, Proteobacteria bacterium CAG 139, Muribaculum intestinale, *Parasutterella Excrementihominis*	*WKY Control versus SHRSP Control:* *WKY Control:* 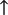 *Mucispirillum schaedleri, Oscilibacter_sp_1_3, Bacteroides vulgatus, B. uniformis, Escherichia coli, Parabacateroides goldsteinii, Akkermania Municiniphila* *SHRSP Control:* 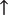 *Proteobacteria bacterium CAG 139, L. johnsonii, Lactobacillus murinus, A. celatus, Adlercreutzia equolifaciens, Bifidobacterium animalis, Bifidobacterium pseudolongum, Turicimonas muris, Muribaculum intestinale, Parasutterella Excrementihominis*
Yang et al., 2022	SHR	Antibiotics + quinapril	Enhanced BP lowering effect of antibiotics + quinapril	*SHR ± quinapril versus SHR ± quinapril ± Antibiotics:* 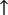 Lachnospiraceae, *Ruminococcus, Prevotella, Oscillibacter,* Ruminococcaceae UCG_014, Lachnospiraceae UCG_006, *Coprococcus 3, Coprococcus 2. Faecalibaculum, Desulfovibrio, Oscillospira*	NA
Shi et al., 2022	SHRSP	Genetic	SHRSP had elevated SBP at age 8 weeks	*SHRSP versus WKY* 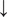 Firmicutes, Deferribacterota, *Oscillibacter* 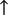 Bacteroidota, Verrucomicrobiota, Proteobacteria, *Akkermansia, Allobaculum, Parasutterella*	NA
Wu et al., 2022	SD rats	Effect of captopril in DOCA-induced HTN	SBP was significantly decreased by CAP treatment in DOCA group	*DOCA:* 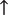 *Escherichia_Shigella, Eubacterium nodatum group, Ruminococcus_2*	*SHAM:* 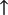 *Staphylococcus*, *Helicobacter*, *Candidatus Saccharimonas* and *Mucispirillum* and genera *Ruminococcaceae UCG007* and *Peptococcus* *DOCA ± CAP:* 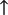 *Bifidobacterium*, *Victivallis*, *Akkermansia*, *Aerococcus, Blautia, Tyzzerella_3* and *Hydrogenoanaerobacterium* 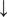 Proteobacteria, Cyanobacteria
Wang et al., 2022	SD rats	Cold (4 degree)-induced HTN	Cold expose for 6 weeks induced HTN	*Cold-exposed group:* 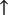 Bacteroidetes, *Prevotella 1, Quinella, Butyricimonas, Peptococcus, Rothia, Senegalimassilia* 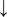 Firmicutes	*Control group:* 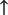 *Lactobacillus, Lachnospiraceae UCG-008*, *Ruminococcaceae UCG-013*, *Pasteurella*, *Lachnospiraceae XPB1014* group, *Ruminococcaceae UCG-010*, *Coprococcus 3*, *Lachnospira*, *Papillibacte*, *Anaerovorax, Lachnospiraceae NK4B4* group and *Acetitomaculum*
Zheng et al., 2022	Wistar rats	HSD	HSD-induced HTN	*High salt:* 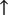 Bacteriodetes, Tenericutes*, B. animalis* 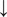 Firmicutes *Unique to HSD group* *Armatimonadetes, Deinococcus-Thermus, FBP, Planctomycetes, Pasteurella multocida, Dubosiella*	*Low salt:* 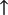 *Ruminococcus_2, Butryricoccus, Lactobacillus acidophilus* 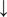 *Allobaculum* *Unique to low salt group* Chloroflexi, Cyanobacteria, *Eubacterium plexicaudatam*
Hsu et al., 2022	SD rats	Maternal Tryptophan free diet (TF)	Offspring exposed to maternal TF diet had elevated BP	*TF diet group:* 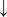 Verrucomicrobia, *Romboustia*, *Akkermansia*, *Ruminococcaceae_NK4A214*, *Roseburia*	NA
Mei et al., 2022	DSS and congenic strain	HSD	Oral Actinobacteria and skin Cyanobacteria are associated with lowering BP	*Congenic strains:* *Skin:* 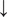 Cyanobacteria in RNO5 female *Oral* **:** 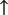 Actinobacteria in RNO10 in both sexes	NA
Avery et al., 2022	C57BL/6 J mice	Germ-Free until 4 weeks and then colonization and Angiotensin (Ang) II treatment	Ang II-induced HTN	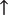 Bacteroidetes 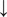 Actinobacteria, *Akkermansia municiniphila,*	NA

**Table 2.** The association observed between human hypertension, gut microbiota and various interventions

References	Model	Intervention	Taxa positively associated with BP	Taxa negatively associated with BP
Munukka et al., 2012	74 premenopausal women and 11 healthy females	NA	*Genera* **:** *Proportion of Eubacterium rectale- Clostridium coccoides* (compared to nonmetabolic disorder group and normal-weight women group)	NA
Queipo-Ortuño et al., 2012	10 healthy male volunteers	Red wine polyphenols and ethanol	*Genera* **:** *Enterococcus, Prevotella, Bacteroides, Bifidobacterium, Bacteroides uniformis, Eggerthella lenta, Blautia coccoides-E. rectale groups*	NA
Gomez-Arango et al., 2016	86 overweight versus 119 obese women	16 weeks gestation	NA	*Genera* **:** *Blautia, Odoribacter*, families Odoribacteraceae Clostridiaceae Christensenellaceae. (More blood pressure in obese women)
Li et al., 2017	41 healthy controls, 56 pre-hypertensives, 99 primary hypertensives	Fecal microbiota transplant	*Genera* **:** *Prevotella, Klebsiella, Porphyromonas, Actinomyces, Desulfovibrio, Fusobacterium*	*Genera* **:** *Bacteroides, Faecalibacterium, Oscillibacter, Roseburia, Bifidobacterium, Coprococcus, Butyrivibrio, Clostridium, Enterococcus, Blautia*
Yan et al., 2017	60 primary hypertensives and 60 controls	NA	*Genera* **:** *Klebsiella, Clostridium, Streptococcus, Parabacteroides, Eggerthella, Salmonella* *Phylum* **:** Proteobacteria	*Genera* **:** *Faecalibacterium prausnitzii, Roseburia* and *Synergistetes* *Phylum* **:** Actinobacteria
Wilck et al., 2017	12 healthy males	Dietary salt	NA	*Genera* **:** *Lactobacillus* spp.
de la Cuesta-Zuluaga et al., 2018	441 men and women	Fecal SCFA excretion	*Genera**:*** *Enterobacter hormaechei, Haemophilus parainfluenzae, Streptococcus, SMB53*	NA
Kim et al., 2018	22 hypertensives and 18 reference cohorts	NA	*Genera* **:** *Alistipes finegoldii, Dorea, Alistipes indistinctus*	*Genera* **:** *E. rectale, Bacteroides thetaiotaomicron, Klebsiella, Burkholderiales bacterium, Burkholderiales noname, Paraprevotella xylaniphila, Bacteroides salyerside, Veillonella, Paraprevotella clara, Ruminococccus callidus*
Liu et al., 2018	94 hypertensives and 94 healthy controls	NA	*Genera* **:** *E. rectale* *Phylum* **:** Firmicutes	*Genera* **:** *B. thetaiotaomicron, Bifidobacterium*
Jackson et al., 2018	756 hypertensives and 1,790 controls	Prescription medication	*Family* **:** Lactobacillaceae, Streptococcaceae, Enterococcaceae	*Family* **:** Dehalobacteriaceae, Christensenellaceae, Oxalobacteraceae, Rikenellaceae, Clostridiaceae, Anaeroplasmataceae, Peptococcaceae
Ried et al., 2018	49 participants with uncontrolled hypertension	Kyolic aged garlic extract supplement	NA	*Genera* (increased in garlic supplement group compared to placebo): *Lactobacillus* *Clostridia* species *Genera* (increased in placebo compared to garlic supplement group): *Faecalibacterium prausnitzi*
Han et al., 2018	99 nontreated hypertensives, 56 pre-hypertensives and 41 normotensives	NA	*Genera* **:** *Prevotella copri* in HTN and pre-HTN group Cronobacter phage CR3 in pre-HTN Cnaphalocrocis medinalis granulovirus in HTN group	Streptococcus virus phiAbc2, Salmonella phage vB SemP Emek, Mycobacterium phage Toto
Dan et al., 2019	67 normotensives 62 hypertensives	NA	*Genera* **:** *Acetobacteroides*, *Alistipes, Bacteroides, Barnesiella, Christensenella, Clostridium sensu stricto*, *Cosenzaea, Desulfovibrio, Dialister, Eisenbergiella, Faecalitalea, Megasphaera, Microvirgula, Mitsuokella, Parabacteroides, Proteiniborus, Terrisporobacter*	*Genera* **:** *Anaerotruncus, Prevotella, Oscillibacter, Butyricimonas, Acetobacteroides, Acidaminobacter, Adlercreutzia, Anaerotruncus, Asteroleplasma, Bulleidia, Cellulosilyticum, Clostridium III, Clostridium IV, ClostridiumXlVa, Coprobacter, Enterococcus, Enterorhabdus, Flavonifractor, Gemmiger, Guggenheimella, Intestinimonas, Lachnospiracea_ incertae_sedis, Lactivibrio, Lactobacillus, Macellibacteroides, Marvinbryantia, Olsenella, Paraprevotella, Parasutterella, Phascolarctobacterium, Prevotella, Romboutsia, Ruminococcus, Sporobacter, Sporobacterium, Sutterella, Vampirovibrio, Veillonella, Victivallis*
Sun et al., 2019	183 hypertensives and 346 normotensives.	NA	*Genera* **:** *Robinsoniella, Catabacter* *Family* **:** Veillonellacaeae	*Genera* **:** *Sporobacter, Anaerovorax, Ruminococcus* *Family* **:** Ruminococcaceae
Li et al., 2019b	63 hypertensives with treatment-naïve hypertension, 104 hypertensive patients undergoing antihypertensive treatment, 26 normal bp patients with hyperlipidemia, 42 healthy controls	NA	*Genera* **:** *Megamonas, Megasphaera, Lactococcus, Alistipes, Subdoligranulum*	*Genera* **:** *Clostridium sensu stricto 1, Romboutsia, Erysipelotrichaceae UCG.003, Ruminococcus 2, Intestinibacter*
Mushtaq et al., 2019	50 patients with grade 3 hypertension, 30 healthy controls	NA	*Genera* **:** *Prevotella_9, Megasphaera, Parasutterella, Escherichia- Shigella, Phascolarctobacterium faecium*	*Genera* **:** *F. prausnitzii, B. uniformis*
Huart et al., 2019	38 hypertensives, 7 borderline and 9 normotensives	21 hypertensives under antihypertensive medication	*Genera* **:** *Clostridium sensu stricto 1*	*Genera* **:** *Ruminococcaceae_ge_ DQ807686, Clostridiales_ge_16S_ OTU1343*
Bellikci-Koyu et al., 2019	12 Kefir consuming group and 10 unfermented milk consuming group. All hypertensives	Kefir and unfermented milk consumption	*Phylum* **:** Bacteroidetes	*Genera* **:** *Lactobacillus* *Bifidobacterium* spp *Phylum* **:** Actinobacteria Firmicutes Verrucomicrobia
Ferguson et al. n.d.	39 subjects with normal salt intake and 93 with high sodium intake	Dietary salt	*Genera* **:** *Prevotella, Bacteroides* *Family* **:** Ruminococcaceae *Phylum* **:** Firmicutes, Proteobacteria	NA
Verhaar et al., 2020	1,937 hypertensives and 2,735 normotensives	Fecal SCFAs	*Genera* **:** *Klebsiella* spp., *Streptococcus*	*Genera* **:** *Roseburia* spp., *Reseburia hominis, Ruminococcaceae, Clostridium sensu stricto 1, Romboutsia, Enterorhabdus*
Shah et al., 2020	ABO study (Enterotype 1; *n* = 53 and Enterotype 2; *n* = 78) and Fair study (*N* = 29)	Dietary soy	*Genus* **:** *Prevotella*, *Dialister* *(in Enterotype 1)*	NA
Palmu et al., 2020	3,291 hypertensives and 3,662 normotensives	Dietary sodium	*Genera* **:** *Anaerostipes, Anaerotruncus, Bacteroides, Blautia, Citrobacter, Colinsella, Coprobacillus, Coprococcus, Dielma, Dorea, Eisenbergiella, Enterobacter, Erysipelatoclostridium, Faecalitalea, Fournierella, Holdemania, Intestinibacter, Kluyvera, Lactococcus, Megasphaera, Phascolarctobacterium, Ruthenibacterium, Mitsuokella, Paraprevotella, Sanguibacteroides, Sutterella, Turicibacter, Acidaminococcus, Actinomyces, Lactobacillus salivarius*	*Genera* **:** *Lactobacillus paracasei, Adlercreutzia, Alloprevotella, Anaerotruncus, Coprobacillus, Faecalicoccus, Fournierella, Hungatella, Parasutterella, Prevotella, Sellimonas, Senegalimassilia, Solobacterium, Tyzzerella*
Chang et al., 2020	27 Preeclampsia (PE) and 36 healthy pregnant control	NA	*Genera (in PE):* *Enterobacter, Escherichia_Shigella* *Phylum* **:** Proteobacteria	*Genera (in PE):* *Blautia, Eubacterium_rectale, Eubacterium_halii, Streptococcus, Bifidobacterium, Collinsella, Alistipes, Subdoligranulum* *Phylum* **:** Firmicutes
Tindall et al., 2020	42 cardiovascular risk adults	Standard western diet run-in and isocaloric study diets	*Genera* **:** After walnut diet (relative to standard western diet) characterized by an elevated blood pressure, LDL cholesterol and BMI *Roseburia, Eubacterium eligensgroup, Lachnospiraceae UCG001, Lachnospiraceae UCG004, Leuconostocaceae* *Relative to walnut fatty acid-matched diet* *Gordonibacter* After the walnut fatty acid-matched diet (relative to standard western diet) *Roseburia*, *E. eligensgroup* After the oleic acid replaces α-linolenic acid diet (relative to standard western diet) *Clostridialesvadin BB60group*	*Genera* **:** After the whole walnut diet *Lachnospiraceae* (inversely correlated with blood pressure)
Silveira-Nunes et al., 2020	48 hypertensives and 32 normotensives	NA	*Genera* **:** *L. salivarius, Eggerthella, Bacteroides plebeius*	*Genera* **:** *Roseburia faecis, F. prausnitzii, Fusobacterium, Coprococcus*
Takagi et al., 2020	54 controls, 97 hypertensives 96 hyperlipidemia and 162 type 2 diabetes mellitus	NA	*Genera* **:** *Collinsella* *Bifidobacterium* *Phylum* **:** Actinobacteria	*Phylum* **:** Bacteroidetes
Capper et al., 2020	36 healthy participants (19 beetroot group and 17 control group)	Whole beetroot consumption in older population	*Genera* **:** *Prevotella_9* *Phylum* **:** Bacteroidetes	*Genera* **:** *Alistipes,* *Faecalibacterium, Akkermansia* *Christensenellaceae_R-7 group*
Calderón-Pérez et al., 2020	29 nontreated hypertensives and 32 normotensives	NA	*Genera* **:** *Bacteroides coprocola, B. plebeius* and genera of *Lachnospiraceae*	*Genera* **:** *Ruminococcaceae NK4A214, Ruminococcaceae_UCG-010, Christensenellaceae_R-7, F. prausnitzii, Roseburia hominis*
Wan et al., 2020	First day versus 2 weeks versus 6 weeks postpartum milk from 117 mothers	Mother’s milk	NA	*Lactobacillus* is lower in 2 weeks milk in gestational pre-hypertensive mothers.
Louca et al., 2021	397 hypertensives and 474 normotensives	NA	NA	*Genera* **:** *Ruminiclostridium 6*
Wang et al., 2021	93 hypertensives and 15 healthy controls	Effect of electroacupuncture in healthy and HTN group	*Genera* **:** *Escherichia-Shigella* Firmicutes/Bacteroidetes lowered after treatment	*Genera* **:** *Blautia*
Liu et al., 2021b	13 primary aldosteronism (PA) patients, 26 sex-matched primary hypertensives, and 26 sex-matched healthy controls	PA and PHTN patients received antihypertensive medications before recruitment.	*Genera* **:** *Megamonas,* *Sutterella* *Lactobacillus,* *Enterococcus* *Bacillus,* *Bifidobacterium, Phascolarctobacterium, Pseudomonas, Weissella, Ruminococcus gnavus group, Pediococcus, Acinetobacter, Lactococcus, Akkermansia, Alloprevotella, Staphylococcus, Wolbachia, Halomonas, Bradyrhizobium*	*Genera* **:** *Faecalibacterium, Subdoligranulum, Roseburia, Coprococcus,* *Blautia, Ruminococcus, Agathobacter, Alistipes, Adlercreutzia, Paraprevotella, Erysipelotrichaceae UCG 003* *Christensenellaceae R-7 group* *Eubacterium ventriosum group* *E. eligens group* Firmicutes/Bacteroidetes ratio *Phylum* **:** Proteobacteria *Family* **:** Lachnospiraceae
Wan et al., 2021	300 healthy controls, 300 hypertensives and 300 coronary heart disease patients	NA	*Genera* **:** Enterobacteriales *Escherichia* *shigella*	*Genera* **:** Acidaminococcaeceae *Phascolarctobacterium* *Phylum* **:** Bacteroidetes, Bacteroidia
Zhong et al., 2021	73 hypertensives and 187 normotensives	Washed microbiota transplant from control to HTN	*Genera* **:** *Parasutterella. Solobacterium*	*Genera* **:** *Senegalimassilia*
Calderón-Pérez et al., 2021	29 hypertensives versus 32 normotensives	Phenolic compound (sources such as coffee, olive fruits and vegetables)	*Genera* **:** *Bacteroides* *coprocola* *Bacteroides* *plebeius*	*Genera* **:** *Ruminococcaceae NK4A214,* *Ruminococcaceae UCG-010,* *Christensenellaceae R-7,* *F. prausnitzii*
Joishy et al., 2022	37 normotensives and 34 hypertensives	NA	*Genera* **:** *Prevotella* (different ASVs)*, Megasphaera, Butyricoccus, Prevotellaceae, Faecalibacterium, Lachnoclostridium, Howardella and g-UCG04*	*Genera* **:** *Prevotella (*other ASVs*), Alloprevotella, Streptococcus*

## From GWAS to PRS for HTN

**Figure 1.**
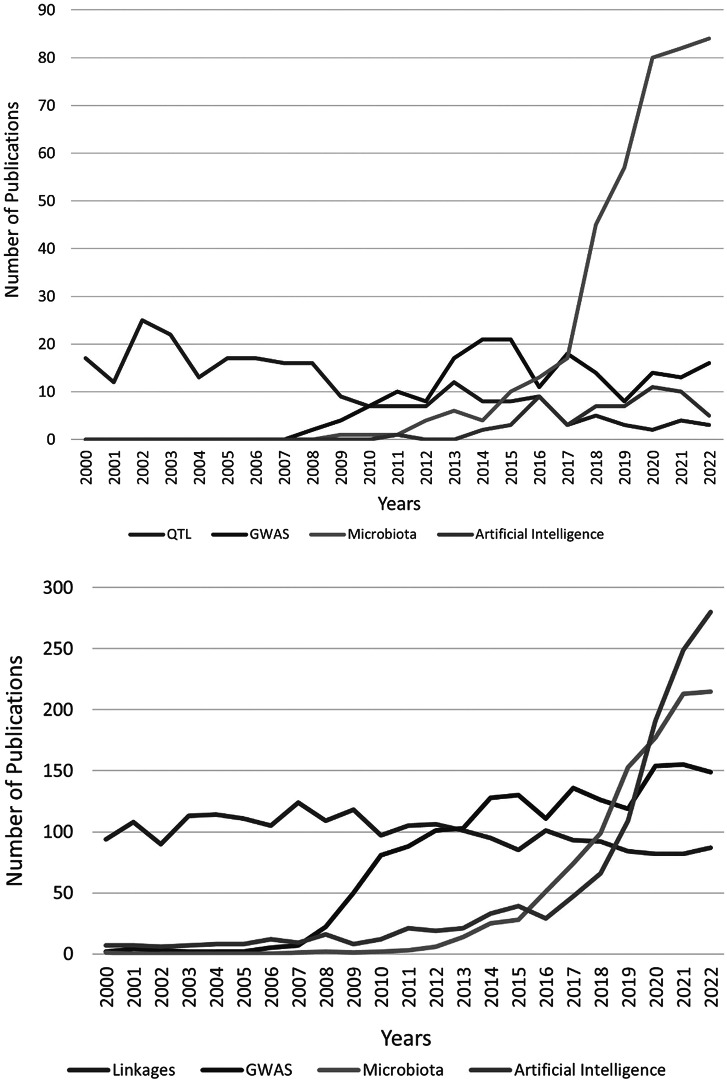
(a) The numbers of PubMed publications (2000–2022) related to quantitative trait locus (QTL), genome-wide association studies (GWAS), microbiota, artificial intelligence in rats and mice hypertension. The search keywords were QTL, hypertension, rats, mice, GWAS, microbiota and artificial intelligence. (b) The numbers of PubMed publications (2000–2022) related to linkage, genome-wide association studies (GWAS), microbiota and artificial intelligence in human hypertension. The search keywords were linkage, hypertension, humans, GWAS, microbiota and artificial intelligence.

Genome-wide association studies (GWAS) aim to analyze genetic variants across genomes to detect associations with complex traits (Dehghan, 2018). GWAS for HTN began in 2007 with the first report of associations in the Wellcome Trust Case Control Consortium (Burton et al., 2007). GWAS for HTN soon outpaced all linkage analyses in humans (Figure 1b). Even so, the collective effect of all BP loci identified through GWAS accounts for ~3.5% of BP variance (Manolio et al., 2009; Sung et al., 2018). This begs the question: ‘What is the expectation from continued investments in GWAS for clinical management of HTN?’ From the perspective of disease risk prediction, continued research in GWAS for HTN is essential for developing, defining and refining the predictive power for HTN using a genomic index, which is known as PRS (Choi et al., 2020; Lewis and Vassos, 2020; Padmanabhan and Dominiczak, 2021). It is computed as the sum of an individual’s genome-wide genotype that is weighted by corresponding genotype effect size estimates (or *Z* scores) generated from a relevant GWAS data (Lewis and Vassos, 2020). Although PRSs often explain only a small portion of trait variance, their link with genetic liability, the single biggest source of phenotypic variation, has rendered PRS as an attractive prediction tool in biomedical research (Choi et al., 2020). PRS is used to assess shared etiologies between phenotypes and to investigate the clinical applicability of genetic information for complex diseases (Choi et al., 2020). Previously, a PRS constructed utilizing genome-wide important single nucleotide polymorphisms from GWAS for BP showed a significant relationship with heart failure, left ventricular mass, coronary artery disease and stroke (Studies, 2011; Ference et al., 2014). Currently, there is considerable excitement in the field for developing reliable PRS for HTN as evident from multiple reports of PRS indices from different cohorts (Steinthorsdottir et al., 2020; Sapkota et al., 2021; Sato et al., 2021; Fujii et al., 2022; Parcha et al., 2022; Quintanilha et al., 2022). Recently, Weng et al. (2022) included 391,366 participants from the UK Biobank database and established a PRS for HTN assessing the combined effect of genetic susceptibility and air pollution on incident of HTN. They demonstrated that long-term exposure to air pollution is associated with increased risk of HTN particularly in individuals with high genetic risk (Weng et al., 2022). Another study from Finland revealed that a BP (systolic and diastolic) PRS could predict HTN in the FINRISK cohort, a Finnish population survey on risk factors on chronic, noncommunicable diseases (Vaura et al., 2021, https://thl.fi/en/web/thl-biobank/). This study highlights the potential of PRS as a predictive tool that may be better than the established clinical risk factors for the prediction of HTN (Vaura et al., 2021). But both studies are limited by their reliance on genetic data from European ancestries, which could limit the predictive power of a PRS in other populations. With the availability of recent, large multi-ethnic and non-European GWAS of BP phenotypes, such as those from the Million Veteran Program, the UK Biobank and Biobank Japan (Kanai et al., 2018; Giri et al., 2019), PRS predictions are now expanded to other demographics, which is a promising outlook for the construction of multi-ethnic PRS for HTN risk prediction (Cavazos and Witte, 2021). More recently, the Trans-Omics in Precision Medicine Initiative program (Stilp et al., 2021; Taliun et al., 2021) reported the assessment of PRS for HTN across major U.S. demographic segments. This included African Americans, Hispanic/Latino Americans, Asian Americans and European Americans in the assessment of PRS associations with HTN across the lifespan. The final HTN-PRS was compared with incident outcomes in the Mass General Brigham Biobank as well as with Multi-ethnic Independent Biobank that included 40,201 subjects, leading to associations that supported the links between PRS and HTN. The resulting PRS was also predictive of an elevated risk of type 2 diabetes, chronic renal disease, coronary artery disease and ischemic stroke (Kurniansyah et al., 2022). Based on these results, Kurniansyah et al. (2022) proposed a new approach for tuning parameters for PRS construction including optimization of the coefficient of variation of the effect size estimates and combining PRS based on GWAS of multiple BP phenotypes into a single PRS. Collectively, the next phase of GWAS in HTN should focus on prediction rather than treatment, the implementation of which will depend on the accuracy for applicability of a PRS for HTN in a global setting. To this end, the ‘All of Us’ research program in the United States is enrolling a million individuals from diverse populations for building a repository that includes genomic data, along with variables such as lifestyle, socioeconomic factors, environment and biological factors (All of Us Research Program Investigators, 2019). The United States is a melting pot of diverse populations from around the world. It is therefore particularly interesting to explore this database for further enhancing the power of PRS for HTN.

## Limitations for PRS-based predictions for HTN

Despite the promise and potential of PRS for HTN, there are clear barriers for its application in a clinical setting. One of the main concerns is the environmental component, which has larger effects than the genetic component on BP and may skew the prediction scores. Additionally, PRS analyses are not well-standardized and may lead to faulty interpretations (Choi et al., 2020). Thus, the focus must move from association with case–control status to individualized PRS for enhancing disease prediction (Lewis and Vassos, 2020). Additionally, absolute risks for the disease should be converted from relative risks that compare people across the PRS continuum with a control group (Torkamani et al., 2018; Sugrue and Desikan, 2019). When using PRS for HTN prediction, management and treatment, it is also required to rigorously differentiate between essential HTN and secondary HTN. Finally, as is the case with all diseases, there are ethical concerns regarding the application of PRS for HTN, which may escalate health inequities (Minari et al., 2018; Martin et al., 2019; Vaura et al., 2021).

## Progress beyond GWAS: What are we missing?

As mentioned above, the premise of using PRS alone for HTN lacks power because of the environmental factors contributing to its etiology. In this context, it is important to note that a prominent, previously unknown, and relatively recent factor identified as contributing to BP regulation is the composition of gut microbiota. As shown in Figure 1a,b, the numbers of studies on microbiota and HTN is sharply rising in both animal models and humans. Interestingly, the sheer numbers of such studies currently surpasses that of GWAS studies, indicating its importance. In the following sections, we review these studies and propose that the inclusion of microbiota signatures and their functional readouts along with the genetic makeup of the host may enhance the power of PRS for HTN.

## Gut microbiota and HTN

A large body of evidence has emerged in the last decade supporting the role of the microbiota in BP regulation. Our group has been at the forefront of this research. In 2010, it was shown that knockout of toll-like receptor 5 (Tlr5) in mice resulted in elevated BP (Vijay-Kumar et al., 2010). Tlr5 is a receptor for the bacterial protein flagellin, suggesting a link between gut microbiota and HTN. However, the major focus of this report was on metabolic syndrome, of which BP is a hallmark. The first evidence for a direct link between gut microbiota and BP regulation in a genetic model of HTN was reported in 2015 in Dahl salt-sensitive (DSS) rat (Mell et al., 2015). Shortly thereafter, an association between gut dysbiosis and HTN in spontaneously hypertensive rats (SHR), angiotensin II-induced hypertensive rats, sleep apnea-induced hypertensive rats (Lloyd et al., 2015) and hypertensive humans were reported (Yang et al., 2015). Since these initial groundbreaking reports, multiple publications have demonstrated associations of gut microbiota with BP regulation in animal models and humans (Tables 1 and 2).

One question of assessing microbiota composition in rats is whether they are translationally relevant for humans. Human gut microbiota composition is more similar to rats than to mice (Flemer et al., 2017), although this question is evolving with continued development of sequencing methods. Here, we access the commonalities in taxonomic rearrangements occurring during HTN in rats and humans. One of the themes emanating from BP association studies using rat as model organism is the application of the Firmicutes/Bacteroidetes (F/B) ratio in assessment of gut dysbiosis in HTN. Increased F/B ratio is regarded as a marker of gut dysbiosis and is consistently reported both in genetic and induced hypertensive rat models including the SHR (Yang et al., 2015; Hsu et al., 2020; Li et al., 2020), DSS rats (Mell et al., 2015; Waghulde et al., 2018) high-fat diet fed rats (Hsu et al., 2019), N^G^-nitro-l-arginine methyl ester (l-NAME) treated rats (Robles-Vera et al., 2018) and angiotensin II induced HTN rats (Yang et al., 2015). In further support, normalizing the F/B ratio by administration of the anti-inflammatory antibiotic, minocycline, alleviated angiotensin II-induced HTN (Yang et al., 2015). This direct relationship between F/B ratio and BP has also been documented in various mouse models (Marques et al., 2017; Toral et al., 2018). Similarly, human studies (Mushtaq et al., 2019; Silveira-Nunes et al., 2020; Joishy et al., 2022) also support a direct relationship between F/B ratio and BP. In contrast, cold-induced HTN (Wang et al., 2022) is one of the rare contexts wherein F/B ratio was not altered significantly. Nevertheless, more robust markers of gut dysbiosis should be developed to posit strong correlation between decreased microbial diversity and HTN.

Beyond the F/B ratio, remodeling of the overall composition of gut microbiota has been documented in the context of HTN. For example, enrichment in gut bacterial lactate producers such as *Streptococcus* and *Turicibacter* (Yang et al., 2015; Toral et al., 2019b; Robles-Vera et al., 2020b) and depletion of butyrate producers such as *Coprococcus,* and *Pseudobutyrivibrio* (Yang et al., 2015; Durgan et al., 2016) are reported in hypertensive rodents. Importantly, *Streptococcus* and *Coprococcus* are also taxa similarly associated with human HTN (Yan et al., 2017; de la Cuesta-Zuluaga et al., 2018; Palmu et al., 2020; Verhaar et al., 2020), and normalizing abundance of these with minocycline and captopril (Yang et al., 2015; Li et al., 2020) lowered BP, further supporting their associations with BP.

Dietary interventions have also been used to study the relationship between gut microbiota and BP. Dietary salt can modulate the composition of microbiota by depleting the abundance of beneficial microbiota including several Lactobacilli species (Mell et al., 2015; Wilck et al., 2017; Bier et al., 2018; Yan et al., 2020). An association between the depletion of *Lactobacillus* and HTN has also been noted in response to maternal and post-weaning high-fat diet, and it suggested that *Lactobacillus* may be beneficial in curbing developmental HTN (Tain et al., 2018; Hsu et al., 2019). Although the mechanisms remain to be clarified, administration of *Lactobacillus murinus* prevented the expansion of proinflammatory IL-17A-producing CD4^+^ T_H_17 lymphocytes in small intestine, colon, and the splenic lamina propria (Wilck et al., 2017). Data from human studies with *Lactobacillus* are however conflicting, as they may be enriched or depleted in hypertensive patients (Wilck et al., 2017; Palmu et al., 2020; Silveira-Nunes et al., 2020; Wan et al., 2020; Liu et al., 2021b).

Gut metabolities, derived either from gut microbiota or involving both gut microbiota and host is of growing interest in the context of HTN. One such important class of microbial metabolites is short-chain fatty acid (SCFA). SCFAs such as acetate, propionate and butyrate are produced by bacterial fermentation of dietary carbohydrates and have been linked with BP regulation. It is reported that decreased SCFA production and the supplementation of SCFA lowered BP in rat and mouse HTN models, indicating the potential for antihypertensive therapy (Marques et al., 2017; Kim et al., 2018; Bartolomaeus et al., 2019; Robles-Vera et al., 2020c). In deoxycorticosterone acetate (DOCA)-salt-induced HTN, high-fiber diet lowered BP and enriched abundance of gut microbes producing acetate (Marques et al., 2017). Interestingly, higher fecal levels of SCFA were associated with hypertensive individuals compared to normotensives (de la Cuesta-Zuluaga et al., 2018; Huart et al., 2019; Calderón-Pérez et al., 2020). Further, increased fecal SCFA was accompanied by decreased plasma SCFA and depleted butyrate-producing bacteria which suggests dysregulated production of SCFA in HTN condition (Calderón-Pérez et al., 2020). The translational relevance and progress of animal as well as clinical studies in SCFA and BP have resulted in a clinical trial to determine the full efficacy of SCFA to treat HTN (Australian New Zealand Clinical Trials Registry ACTRN12619000916145). This phase II clinical trial used two SCFAs, acetate and butyrate which were supplemented with high-amylose maize, and the patients receiving the treatment showed 24-h BP lowering effect with the increase in gut microbes producing SCFA (Jama et al., 2023), which is another evidence of the promising potential of targeting gut microbiota in HTN treatment. Another notable microbial metabolite is trimethylamine-*N* oxide (TMAO). Trimethylamine is produced by gut microbiota, and subsequently oxidized in the liver to form TMAO. Studies have shown the associations between higher plasma levels of TMAO and CVDs (Koeth et al., 2013, 2019; Wang et al., 2014). In a meta-analysis involving human studies, higher circulating TMAO concentration was positively associated with an increased risk of HTN (Ge et al., 2020). TMAO feeding further increased BP and promoted vasoconstriction in angiotensin II-induced hypertensive mice (Jiang et al., 2021). Besides SCFA and TMAO, there are many microbiota-derived metabolites such as indole, indole-3-acetic acids and secondary bile acids among others, that may have significant roles in BP regulation (Huć et al., 2018; Chakraborty et al., 2020a). The knowledge on the effects of these microbial metabolites is growing, however, the precise underlying working mechanisms remain largely unknown.

## Gut microbiota restructured in HTN: Cause, consequence or adaptation?

While an association between the reprogramming of gut microbiota and HTN is established, whether gut dysbiosis is a cause or a consequence of HTN is an important question to focus on. Initial experiments were designed to address this question by using antibiotics to eliminate endogenous gut microbiota. However, such studies did not provide conclusive evidence for cause or consequence because different antibiotics affected BP differently depending on both the type of antibiotic and the rodent strain (Galla et al., 2018, 2020). More convincing evidence for microbiota to cause a BP effect was obtained using germ-free Sprague Dawley (SD) rats. We showed that these rats which lack microbiota are hypotensive, with a prominent loss of vascular tone (Joe et al., 2020). These findings are the first to clearly demonstrate that the host requires gut microbiota for BP homeostasis and maintenance of vascular tone (Joe et al., 2020). One caveat to these studies is that the model used is not hypertensive. To establish the cause-effect relationship between HTN and gut microbiota, animal models such as germ-free hypertensive rat models can be developed. Such hypertensive germ-free rats will allow for testing the hypothesis that lack of microbiota will render them resistant to HTN. Currently, the lack of germ-free hypertensive rats as tools is a technical barrier to understand whether microbiota cause HTN.

To examine the causality of gut dysbiosis, Adnan et al. (2017) performed gut microbiota cross-transplants between WKY and spontaneously hypertensive stroke-prone rats (SHRSP, a rodent model of HTN associated with high incidence of stroke. They observed that a stable transplant of SHRSP gut microbiota to normotensive WKY recipients, by oral gavage, led to a significant elevation in BP (Adnan et al., 2017). Similar trend was observed in another study after fecal microbiotal transplantation from SHR to WKY (Toral et al., 2019b). As an alternative approach to oral gavage transplants, which involve exposure to antibiotics, Nelson et al. (2021) swapped WKY and SHRSP gut microbiota using a cross-fostering protocol. By fostering newborn rat pups with a dam of the opposite strain, SHRSP rats were populated with a WKY gut microbiota and vice versa. Under these conditions, WKY rats harboring SHRSP gut microbiota developed a significantly elevated BP in adulthood compared to WKY rats with native WKY microbiota. Conversely, adult SHRSP rats harboring the WKY gut microbiota presented with significantly lower BP compared to SHRSP with their native SHRSP microbiota (Nelson et al., 2021). These data signify that initial colonization of gut microbiota is critical and has long-lasting consequences on host pathophysiology.

As highlighted above, research focus has shifted to better understanding the mechanisms of host-microbiota interactions. Emerging studies address molecular mechanisms and demonstrate how BP may be regulated by bacterial metabolites via effects on the aryl hydrocarbon receptor (Natividad et al., 2018; Liu et al., 2021a), and G-protein coupled receptors (Marques et al., 2018; Xu and Marques, 2022) among others, which may impact end organ functions of the kidney, vasculature, brain and heart. Emerging work from Durgan et al. shows that a new mechanism by which signals derived from the gut microbiota (i.e., metabolites, neurotransmitters, endotoxins) may be distributed throughout the host via packaging into outer membrane vesicles (OMVs). These OMVs are lipid-bound vesicles (as known as bacterial liposomes) released from the gut microbiota that are capable of crossing the gut barrier and entering the systemic circulation. Bacterial OMVs can carry a wide range of ‘cargo’ including proteins, lipids, and small RNA, that can be delivered to and exert effects on distant host cells. They have shown that OMVs from the SHRSP microbiota have unique protein and lipid cargo as compared to OMVs from the WKY microbiota. Additionally, they find that SHRSP OMVs gavaged to WKY rats leads to significant elevations in BP (Shi et al., 2021a).

Previous reports showed no gut dysbiosis in pre-hypertensive SHR (Santisteban et al., 2017; Yang et al., 2020), suggesting that gut dysbiosis may arise as a consequence of HTN. However, these studies demonstrated colonic changes in pre-hypertensive SHR indicating a dysregulated gut barrier before developing HTN. Future studies should address this more specifically. Nevertheless, transplant experiments show that gut dysbiosis contributes to HTN and that manipulation of gut microbiota can alleviate HTN, suggesting that gut microbiota could be a potential therapeutic target.

## Gut microbiota as therapeutic targets

There is considerable excitement of targeting gut microbiota for translational applications as evident from ongoing clinical trials for microbiota-guided therapies for HTN (https://clinicaltrials.gov/ct2/results?cond=hypertension&term=minocycline&cntry=&state=&city=&dist=). In preclinical studies, our group recently proposed *Faecalibacterium prausnitzii* as a novel probiotic to attenuate chronic kidney disease (CKD) conditions, following demonstrated depletion of *F. prausnitzii* in CKD patients in eastern and western human hypertensive populations. Importantly, supplementation of *F. prausnitzii* in a CKD mouse model not only ameliorated renal dysfunction, renal inflammation, and the levels of uremic toxins, but also improved gut ecology and intestinal integrity (Li et al., 2022). Since *F. prausnitzii* has also been reported to be depleted in CVD (Jie et al., 2017; Aryal et al., 2020), and CVD and CKD are highly correlated, it is possible that enhancing *F. prausnitzii* could be beneficial for CVD, for which HTN is a major risk factor (Li et al., 2022). Supporting this notion, studies have shown that *F. prausnitzii* is significantly abundant in normotensive compared to hypertensive humans, demonstrating strong correlation of this specific microbe with BP (Yan et al., 2017; Calderón-Pérez et al., 2020). However, in contradiction, *Faecalibacterium* was more enriched in individuals with high BP (Joishy et al., 2022). Therefore, there is a need to directly examine the potential of *F. prausnitzii* in rigorous animal model studies.

In addition to being considered as therapeutic agents, gut microbiota may be involved in the modulation of our responses to antihypertensive medications (Kyoung et al., 2022). The efficacy of angiotensin-converting enzyme (ACE) inhibitors is reportedly modulated by the gut microbiota (Kyoung and Yang, 2022; Yang et al., 2022). Our group has recently demonstrated that quinapril, which is absorbed in the gut and metabolized by esterases in the liver to yield an active metabolite in circulation is prematurely catabolized in the gut by microbiota. This led to reduced availability of the active metabolite, quinaprilatin in circulation, which was associated with reduced BP responses to oral administration of quinalapril (Yang et al., 2022). We further identified that a specific microbiota, *Coprococcus comes*, contains a bacterial form of esterase and may be one of the culprits for the premature quinalapril degradation and reduction in its efficacy as a BP-lowering agent. Interestingly, a higher abundance of *C. comes* is present in the African American hypertensive population (Yang et al., 2022) who are known to respond poorly to ACE inhibitor treatments compared to Caucasian hypertensive patients (Yang et al., 2022). This proof-of-concept study implicates that gut microbiota is a crucial factor defining individualized responses to hypertensive medications that should be addressed in future efficacy studies of antihypertensive drugs.

## Current limitations in microbiome research for HTN

While such physiological studies are clearly important, drawing conclusions about the role of individual microbes in BP regulation, based on 16S analysis alone, can be problematic. The issue stems from the fact that multiple species can carry out the same function (e.g., generate the same metabolite), also referred to as functional redundancy. This redundancy likely contributes to the disparate candidate bacteria identified across hypertensive models and research facilities (Table 1). Another limitation of 16S analysis is that it captures a limited portion of the bacterial genome (Lewis et al., 2021). These limitations can be addressed by sequencing of whole bacterial genomes known as metagenomic sequencing, which is an emerging area in HTN research (Walejko et al., 2018; Shi et al., 2021b). It should be noted that at the current stage, it is a misnomer to use the term ‘microbiome’ until metagenomes are reported. With the advent of rapid and cost-effective technologies, progress in reporting of metagenomes is anticipated to provide a platform for association studies of metagenomes with HTN. Metagenomics however falls short in assessing activity of the identified bacterial genes. Thus, combining metagenomics with an assessment of the functional output from the microbiota (i.e., proteomics, metabolomics, lipidomics) can be especially powerful. A recent study by the Durgan laboratory examined the role of gut dysbiosis in the SHRSP model by combining metagenomics with metabolomics analysis of the cecal bacterial content and the host plasma. While metabolomics revealed significant reductions in cecal and plasma primary and secondary bile acids in the SHRSP, metagenomics pinpointed that specific genes encoding bacterial enzymes involved in bile acid transformation were also reduced in the SHRSP microbiota. Thus, assessing changes in microbiota function will be useful in the development of targeted approaches for the treatment of HTN.

One other limitation in elucidation of functional consequences of host-microbiota interactions is addressing the complexity of such interactions. Gut microbiota and the host have evolved for centuries to live in complete symbiosis. This means that they are mutually dependent for survival and homeostasis. For example, humans are not capable of degrading fiber (Kaoutari et al., 2013; Cockburn and Koropatkin, 2016). Gut bacteria aid the host by fermentation of fiber, thus generating SCFAs which are the main source of energy for the host colonic epithelium (Koh et al., 2016; Baxter et al., 2019). Thus, the reduction of beneficial SCFA-producing bacteria, as seen in human and rodent HTN (Yang et al., 2015; Gomez-Arango et al., 2016; Kim et al., 2018; Yang et al., 2019b; Calderón-Pérez et al., 2020; Overby and Ferguson, 2021), disrupts the symbiotic host-microbiota relationship leading to disease. In return, the bacteria have most likely evolved by adapting and responding to the host, as evidenced by the effects of genetic host manipulations on gut bacterial composition (Yang et al., 2017; Bartley et al., 2018). Recent studies have attempted to address the complexity of host-microbiota interactions in symbiosis and dysbiosis. Of note, a recent study using isotope tracing found that the host can regulate the composition of gut bacteria by allowing the passage of host-circulating metabolites into the gut (Zeng et al., 2022). These host/gut cometabolites were found to be beta-hydroxybutyrate (BHB), lactate and urea, among other, which are preferentially utilized as nutrients by certain bacterial communities. Future studies should investigate how these cometabolites contribute to regulation of gut microbiota eubiosis and how this interaction reflects on BP regulation. We have recently shown that the circulating BHB and gut microbiota are both salt-responsive (Chakraborty et al., 2018). Moreover, we found that circulating BHB was decreased with high salt feeding, and that supplementation with BHB alleviated salt-sensitive HTN, but the contribution of gut microbiota to BHB generation or the potential direct effect of BHB on gut microbiota in BP regulation remains unknown. Thus, gut microbiota may have coevolved with the host to produce, utilize and respond to a variety of the same metabolite-substrate-effectors, reflected in the expression of some of the same genes by the bacteria and the host (Bartley et al., 2018; Yang et al., 2018, 2022; Hsu et al., 2022). Thus, the utilization of combined omics, employed at both microbiota and host levels, will lead to better predictions and targeting of host-microbiota interactions for therapeutics.

In conclusion, as noted through the numbers of studies reported in PubMed, microbiota is an emerging and important research area in HTN, surpassing that of GWAS and QTL studies of HTN (Figure 1a,b). Although research is still in early conception, given that the gut metagenomes co-evolve with the host and are critical for BP regulation, risk prediction for HTN using a PRS may be more informative in combination with new bacterial analysis approaches leading up to a ‘MRS’ that encompass both the metagenomic profiles and the functional bacterial readouts. The groundwork required for accumulating metagenomic signatures is admittedly daunting because of the fluctuating nature of microbiota, but collecting these data is important for its ultimate convergence with PRS for enhancing predictive strategies for HTN. Such an endeavor demands intense computational analyses that may only be addressable with AI strategies.

## An application of AI and machine learning in HTN research

AI refers to methods for transferring human intellect to computers that can stimulate human learning and thought processes by using sophisticated algorithms and powerful computing capacity to process large amounts of data (Chaikijurajai et al., 2020; Tsoi et al., 2021). Machine learning (ML) and deep learning (DL) are the two subclasses of AI (Tsoi et al., 2021). ML finds the association between the provided training datasets with variables and then performs predictive analyses on the new sets of data (Tsoi et al., 2021). ML is further classified into supervised and unsupervised learning (Chaikijurajai et al., 2020). Supervised ML relies on the labeled input–output paired data which is then used for the prediction of known output (Cheng et al., 2011). It employs a variety of methods, including neural networks, support vector machines, random forest and naive Bayes (Cheng et al., 2011). On the other hand, unsupervised ML employs unlabeled datasets to predict unknown outputs by detecting underlying patterns or correlations among the variables (Cheng et al., 2011; Chaikijurajai et al., 2020). The principal use of DL is pattern recognition, such as in voice and image analysis (Chaikijurajai et al., 2020).

AI is increasingly being used in human HTN research (Figure 1b). Recent studies have shown how AI has the ability to reduce the worldwide burden of HTN and promote the development of HTN-related precision medicine (Golino et al., 2014; Ye et al., 2018; Lacson et al., 2019; Kanegae et al., 2020; López-Martínez et al., 2020; Soh et al., 2020; Schrumpf et al., 2021). As a result, the main goal of these investigations is to enhance the clinical management of HTN. Persell et al. conducted a randomized clinical trial of 297 persons with uncontrolled HTN to examine the impact of AI, smartphone coaching apps monitoring systolic BP and HTN-associated behavior. At the 6-month follow-up, the researchers did not discover decreased BP, but they did create a space for the possibility of different treatment effects among age subgroups (Persell et al., 2020). Pan et al. (2019) coupled auscultatory waveforms data with ML to measure BP from Korotkoff sound recordings and examine the impact of movement disturbance on BP regulation. Among 40 healthy volunteers, their brand-new DL-based automatic BP measurement instrument showed encouraging accuracy in BP monitoring both when resting and not resting (Pan et al., 2019). In 965 participants, Li et al. employed ML to identify genetic and environmental risk factors for HTN. To identify risk factors for HTN in the Northern Han Chinese population, they created two separate models for systolic BP (composed of age, body mass index, waist circumference, exercise [times per week], parental history of HTN [either or both], and 1 SNP (rs7305099)) and diastolic BP {composed of weight, drinking, exercise [times per week], triglyceride, parental history of HTN [either or both] and 3 SNPs (rs5193, rs7305099, rs3889728)} with an AUC (area under the curve) of 0.673 and 0.817 for the systolic BP and diastolic BP models respectively (Li et al., 2019a). Future use of these AI/ML technologies to HTN may be combined to create a ‘clinical risk score’ (CRS).

To investigate the multifactorial causes of high BP, Louca et al. recently combined environmental, dietary, genetic, metabolite, biochemical and clinical data from two different cohorts. Then they applied the ML XGBoost algorithm to this multimodal domain. They included 4,863 TwinsUK subjects for the study and used 2,807 subjects from the Qatari Biobank to validate their findings. They discovered 30 overlapping features between the two groups, including age, BMI, sex, dihomo-linolenate, urate, *cis*-4-decenoyl cartinine, lactate, glucose, cortisol, chloride, histidine and creatinine to be associated with HTN. These BP biomarkers are crucial for prioritizing mechanistic investigations and for finding effective novel therapies for HTN (Louca et al., 2022). Although this research examined a number of significant CRS and PRS domains to pinpoint the critical elements involved in the regulation of BP, gut microbiota features, which are crucial for building MRS, were not taken into account. Nakai et al. in their recent study performed the first gut microbiome multisite study involving 70 human subjects with HTN and without HTN. The authors combined ML with microbiome pathway analysis and reported differential microbial gene pathways between hypertensive and normotensive participants despite similar gut microbiota profile (Nakai et al., 2021).

As one of the initial steps toward application of AI/ML in development of a MRS for HTN, our group had recently interrogated if the composition of microbiota may be used to classify patients with or without CVDs (Aryal et al., 2020). Due to the lack of information on the status of HTN in the American Gut Project, we resorted to classification of a broader group of patients. Using the top operational taxonomic unit features obtained from fecal 16S ribosomal RNA sequencing data of 478 CVD and 473 non-CVD human subjects, random forest, a supervised ML algorithm, was able to correctly classify between patients with CVD and without CVD, with an AUC value of 0.70. It denotes the prospective capacity of ML for case and control distinction (Aryal et al., 2020). Considering the wide range of variability in binning CVD as a single phenotype, an AUC of 0.7 further signifies that microbiota contribute to CVD, and that an association between disease and microbiota can be identified using AI. To move closer to the eventual objective of creating MRS for HTN, such data are required in the context of HTN.

**Figure 2.**
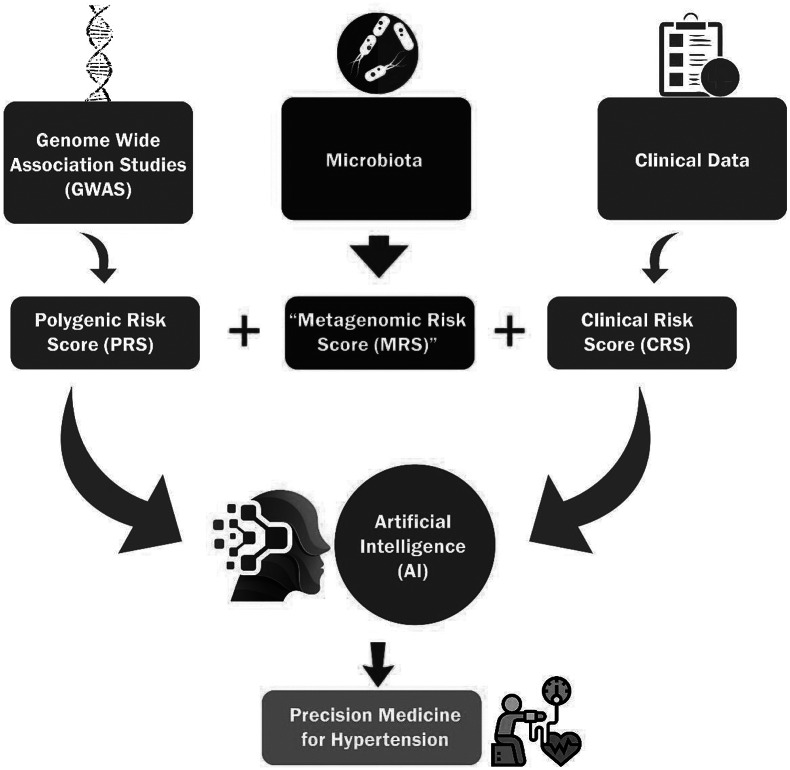
The integration of polygenic risk score, metagenomic risk score and clinical risk score using artificial intelligence is required for the precision medicine in hypertension.

Beyond its usage in healthcare, AI/ML can be used to understand GWAS results by spotting intricate underlying data patterns that make predictions easier. Such methodology improved the prediction of PRS for height, body mass index and diabetes (Paré et al., 2017). Since there exist high-quality GWAS data for HTN, there is a possibility that similar AI/ML methodologies will be merged with CRS and MRS to improve the translational capacities of PRS for HTN (Figure 2).

## Limitations of AI in HTN research

Although the use of AI in HTN has the potential to revolutionize risk prediction, this goal has significant constraints as listed below: (i) There are currently no standards for reporting AI investigations in HTN cases with sufficient rigor. In many publications, for instance, external validation datasets are not used. Very few research articles report the model calibration metrics and, bias brought about by algorithms is typically disregarded (Du Toit et al., 2023). (ii) There is a paucity of open-access databases that provide information on the genotypic and phenotypic characteristics of HTN. (iii) a major current limitation is that large cohort data containing both genomic and microbiota data are lacking. (iv) AI/ML operates in a ‘black box’ (i.e., it is unclear how it does what it does), which is claimed to be the main reason why physicians are reluctant to implement AI technology in clinical practice (Cheng et al., 2011). (v) the interpretability of the AI models, the absence of cause-and-effect reasoning, the capacity to self-monitor errors, and the presence of societal biases are a few more drawbacks (Padmanabhan et al., 2021).

Some solutions for the limitations mentioned above could be to (i) develop easily interpretable AI models which can discern the relationship between the variables contributing to HTN, (ii) promote initiatives for setting up large-scale and rapid data-sharing of large cohort data specifically pertinent to HTN and (iii) development of standardized methodologies to control for rigor of human judgment into the AI systems for determining errors (Padmanabhan et al., 2021).

In conclusion, this review has summarized the mounting evidence that BP is closely correlated with the microbiota, which make up the second-largest genome after the host genome. In light of this borgeoning evidence, we propose exploiting such data for the development of a MRS as a predictive index for HTN. Additionally, we propose using MRS as part of a larger framework that incorporates PRS and CRS to build an AI-based model. Considerable research efforts to generate MRS may serve as a tool to enhance the existing, primarily insufficient predictive capability for the management of HTN.

## Data Availability

Data availability is not applicable to this article as no new data were created or analyzed in this study.

## References

[r1] Abboud FM, Cicha MZ, Ericsson A, Chapleau MW and Singh MV (2021) Altering early life gut microbiota has long-term effect on immune system and hypertension in spontaneously hypertensive rats. Frontiers in Physiology 1920, 752924. 10.3389/fphys.2021.752924 PMC8586697 34777016

[r2] Adnan S, Nelson JW, Ajami NJ, Venna VR, Petrosino JF, Bryan RM and Durgan DJ (2017) Alterations in the gut microbiota can elicit hypertension in rats. Physiological Genomics 49(2), 96–104. 28011881 10.1152/physiolgenomics.00081.2016 PMC5336599

[r3] All of Us Research Program Investigators (2019) The “all of us” research program. New England Journal of Medicine 381(7), 668–676. 31412182 10.1056/NEJMsr1809937 PMC8291101

[r4] Aryal S, Alimadadi A, Manandhar I, Joe B and Cheng X (2020) Machine learning strategy for gut microbiome-based diagnostic screening of cardiovascular disease. Hypertension 76, 1555–1562. 32909848 10.1161/HYPERTENSIONAHA.120.15885 PMC7577586

[r5] Avery EG, Bartolomaeus H, Rauch A, Chen CY, N’Diaye G, ,Löber U, Bartolomaeus TUP, Fritsche-Guenther R, Rodrigues AF, Yarritu A, Zhong C, Fei L, Tsvetkov D, Todiras M, Park JK, Markó L, Maifeld A, Patzak A, Bader M, Kempa S, Kirwan JA, Forslund SK, Müller DN and Wilck N (2022) Quantifying the impact of gut microbiota on inflammation and hypertensive organ damage. Cardiovascular Research. 00, 1–12 10.1093/cvr/cvac121. PMC10262185 35904261

[r6] Bartley A, Yang T, Arocha R, Malphurs WL, Larkin R, Magee KL, Vickroy TW and Zubcevic J (2018) Increased abundance of Lactobacillales in the colon of beta-adrenergic receptor knock out mouse is associated with increased gut bacterial production of short chain fatty acids and reduced IL17 expression in circulating CD4+ immune cells. Frontiers in Physiology 9, 1593. 30483153 10.3389/fphys.2018.01593 PMC6242911

[r7] Bartolomaeus H, Balogh A, Yakoub M, Homann S, Markó L, Höges S, Tsvetkov D, Krannich A, Wundersitz S, Avery EG, Haase N, Kräker K, Hering L, Maase M, Kusche-Vihrog K, Grandoch M, Fielitz J, Kempa S, Gollasch M, Zhumadilov Z, Kozhakhmetov S, Kushugulova A, Eckardt KU, Dechend R, Rump LC, Forslund SK, Müller DN, Stegbauer J and Avery EG (2019) Short-chain fatty acid propionate protects from hypertensive cardiovascular damage. Circulation 139(11), 1407–1421. 30586752 10.1161/CIRCULATIONAHA.118.036652 PMC6416008

[r8] Baxter NT, Schmidt AW, Venkataraman A, Kim KS, Waldron C and Schmidt TM (2019) Dynamics of human gut microbiota and short-chain fatty acids in response to dietary interventions with three fermentable fibers. MBio 10(1), e02566–e02518. 30696735 10.1128/mBio.02566-18 PMC6355990

[r9] Bellikci-Koyu E, Sarer-Yurekli BP, Akyon Y, Aydin-Kose F, Karagozlu C, Ozgen AG, Brinkmann A, Nitsche A, Ergunay K, Yilmaz E and Buyuktuncer Z (2019) Effects of regular kefir consumption on gut microbiota in patients with metabolic syndrome: A parallel-group, randomized, controlled study. Nutrients 11(9), 2089. 31487797 10.3390/nu11092089 PMC6769690

[r10] Bier A, Braun T, Khasbab R, Di Segni A, Grossman E, Haberman Y and Leibowitz A (2018) A high salt diet modulates the gut microbiota and short chain fatty acids production in a salt-sensitive hypertension rat model. Nutrients 10(9), 1154. 30142973 10.3390/nu10091154 PMC6164908

[r11] Biino G, Parati G, Concas MP, Adamo M, Angius A, Vaccargiu S and Pirastu M (2013) Environmental and genetic contribution to hypertension prevalence: Data from an epidemiological survey on Sardinian genetic isolates. PLoS One 8(3), e59612. 23527229 10.1371/journal.pone.0059612 PMC3603911

[r12] Buniello A, MacArthur JAL, Cerezo M, Harris LW, Hayhurst J, Malangone C, McMahon A, Morales J, Mountjoy E, Sollis E, Suveges D, Vrousgou O, Whetzel PL, Amode R, Guillen JA, Riat HS, Trevanion SJ, Hall P, Junkins H, Flicek P, Burdett T, Hindorff LA, Cunningham F and Parkinson H (2019) The NHGRI-EBI GWAS catalog of published genome-wide association studies, targeted arrays and summary statistics 2019. Nucleic Acids Research 47(D1), D1005–D1012. 30445434 10.1093/nar/gky1120 PMC6323933

[r13] Cabrera CP, Ng FL, Nicholls HL, Gupta A, Barnes MR, Munroe PB and Caulfield MJ (2019) Over 1000 genetic loci influencing blood pressure with multiple systems and tissues implicated. Human Molecular Genetics 28(R2), R151–R161. 31411675 10.1093/hmg/ddz197 PMC6872427

[r14] Calderón-Pérez L, Gosalbes MJ, Yuste S, Valls RM, Pedret A, Llauradó E, Jimenez-Hernandez N, Artacho A, Pla-Pagà L, Companys J, Ludwig I, Romero MP, Rubió L and Solà R (2020) Gut metagenomic and short chain fatty acids signature in hypertension: A cross-sectional study. Scientific Reports 10(1), 1–16. 32296109 10.1038/s41598-020-63475-w PMC7160119

[r15] Calderón-Pérez L, Llauradó E, Companys J, Pla-Pagà L, Pedret A, Rubió L, Gosalbes MJ, Yuste S, Solà R and Valls RM (2021) Interplay between dietary phenolic compound intake and the human gut microbiome in hypertension: A cross-sectional study. Food Chemistry 344, 128567. 33203597 10.1016/j.foodchem.2020.128567

[r16] Capper TE, Houghton D, Stewart CJ, Blain AP, McMahon N, Siervo M, West DJ and Stevenson EJ (2020) Whole beetroot consumption reduces systolic blood pressure and modulates diversity and composition of the gut microbiota in older participants. NFS Journal 21, 28–37.

[r17] Cavazos TB and Witte JS (2021) Inclusion of variants discovered from diverse populations improves polygenic risk score transferability. Human Genetics and Genomics Advances 2(1), 100017. 33564748 10.1016/j.xhgg.2020.100017 PMC7869832

[r18] Chaikijurajai T, Laffin LJ and Tang WHW (2020) Artificial intelligence and hypertension: Recent advances and future outlook. American Journal of Hypertension 33(11), 967–974. 32615586 10.1093/ajh/hpaa102 PMC7608522

[r19] Chakraborty S, Galla S, Cheng X, Yeo JY, Mell B, Singh V, Yeoh BS, Saha P, Mathew AV, Vijay-Kumar M and Joe B (2018) Salt-responsive metabolite, β-hydroxybutyrate, attenuates hypertension. Cell Reports 25(3), 677–689. 30332647 10.1016/j.celrep.2018.09.058 PMC6542293

[r20] Chakraborty S, Mandal J, Cheng X, Galla S, Hindupur A, Saha P, Yeoh BS, Mell B, Yeo JY, Vijay-Kumar M, Yang T and Joe B (2020b) Diurnal timing dependent alterations in gut microbial composition are synchronously linked to salt-sensitive hypertension and renal damage. Hypertension 76(1), 59–72. 32450738 10.1161/HYPERTENSIONAHA.120.14830 PMC7289684

[r21] Chakraborty S, Lulla A, Cheng X, McCarthy C, Yeo JY, Mandal J, Alimadadi A, Saha P, Yeoh BS, Mell B, Jia W, Putluri V, Putluri N, Sreekumar A, Wenceslau CF, Kumar MV, Meyer KA and Joe B (2020a) Abstract P238: Bile acid metabolites modulate hypertension. Hypertension 76(Suppl_1), AP238.

[r22] Chang Y, Chen Y, Zhou Q, Wang C, Chen L, di W and Zhang Y (2020) Short-chain fatty acids accompanying changes in the gut microbiome contribute to the development of hypertension in patients with preeclampsia. Clinical Science 134(2), 289–302. 31961431 10.1042/CS20191253

[r23] Chen Y, Zhu Y, Wu C, Lu A, Deng M, Yu H, Huang C, Wang W, Li C, Zhu Q and Wang L (2020) Gut dysbiosis contributes to high fructose-induced salt-sensitive hypertension in Sprague-Dawley rats. Nutrition 75, 110766. 32305658 10.1016/j.nut.2020.110766

[r24] Cheng X, Manandhar I, Aryal S and Joe B (2011) Application of artificial intelligence in cardiovascular medicine. Comprehensive Physiology 11(4), 2455–2466. 10.1002/cphy.c200034 34558666

[r25] Choi SW, Mak TS-H and O’Reilly PF (2020) Tutorial: A guide to performing polygenic risk score analyses. Nature Protocols 15(9), 2759–2772. 32709988 10.1038/s41596-020-0353-1 PMC7612115

[r26] Cockburn DW and Koropatkin NM (2016) Polysaccharide degradation by the intestinal microbiota and its influence on human health and disease. Journal of Molecular Biology 428(16), 3230–3252. 27393306 10.1016/j.jmb.2016.06.021

[r27] Dan X, Mushi Z, Baili W, Han L, Enqi W, Huanhu Z and Shuchun L (2019) Differential analysis of hypertension-associated intestinal microbiota. International Journal of Medical Sciences 16(6), 872. 31337961 10.7150/ijms.29322 PMC6643114

[r28] de la Cuesta-Zuluaga J, Mueller NT, Álvarez-Quintero R, Velásquez-Mejía E, Sierra J, Corrales-Agudelo V, Carmona J, Abad J and Escobar JS (2018) Higher fecal short-chain fatty acid levels are associated with gut microbiome dysbiosis, obesity, hypertension and cardiometabolic disease risk factors. Nutrients 11(1), 51. 30591685 10.3390/nu11010051 PMC6356834

[r29] Dehghan A (2018) Genome-wide association studies. Methods in Molecular Biology 1793, 37–49. 29876890 10.1007/978-1-4939-7868-7_4

[r30] Durgan DJ, Ganesh BP, Cope JL, Ajami NJ, Phillips SC, Petrosino JF, Hollister EB and Bryan RM (2016) Role of the gut microbiome in obstructive sleep apnea-induced hypertension. Hypertension 67(2), 469–474. 26711739 10.1161/HYPERTENSIONAHA.115.06672 PMC4713369

[r169] du Toit C, Tran TQB, Deo N, Aryal S, Lip S, Sykes R, … & Padmanabhan S (2023). Survey and Evaluation of Hypertension Machine Learning Research. Journal of the American Heart Association 12(1) e027896. 10.1161/JAHA.122.027896. 37119074 PMC10227215

[r31] Ference BA, Julius S, Mahajan N, Levy PD, Williams Sr KA and Flack JM (2014) Clinical effect of naturally random allocation to lower systolic blood pressure beginning before the development of hypertension. Hypertension 63(6), 1182–1188. 24591335 10.1161/HYPERTENSIONAHA.113.02734

[r32] Ferguson JF, Aden LA, Barbaro NR, van Beusecum JP, Xiao L, Simons AJ, Warden C, Pasic L, Himmel LE, Washington MK, Revetta FL, Zhao S, Kumaresan S, Scholz MB, Tang Z, Chen G, Reilly MP and Kirabo A (2019) High dietary salt-induced dendritic cell activation underlies microbial dysbiosis-associated hypertension. JCI Insight 5(13), 2019. 10.1172/jci.insight.126241 PMC6629246 31162138

[r33] Flemer B, Gaci N, Borrel G, Sanderson IR, Chaudhary PP, Tottey W, O’Toole PW and Brugère J-F (2017) Fecal microbiota variation across the lifespan of the healthy laboratory rat. Gut Microbes 8(5), 428–439. 28586297 10.1080/19490976.2017.1334033 PMC5628645

[r34] Fujii R, Hishida A, Nakatochi M, Tsuboi Y, Suzuki K, Kondo T, Ikezaki H, Hara M, Okada R, Tamura T, Shimoshikiryo I, Suzuki S, Koyama T, Kuriki K, Takashima N, Arisawa K, Momozawa Y, Kubo M, Takeuchi K, Wakai K and J-MICC Study Group (2022) Associations of genome-wide polygenic risk score and risk factors with hypertension in a Japanese population. Circulation: Genomic and Precision Medicine 15(4), e003612. 35666837 10.1161/CIRCGEN.121.003612

[r35] Galla S, Chakraborty S, Cheng X, Yeo JY, Mell B, Chiu N, Wenceslau CF, Vijay-Kumar M and Joe B (2020) Exposure to amoxicillin in early life is associated with changes in gut microbiota and reduction in blood pressure: Findings from a study on rat dams and offspring. Journal of the American Heart Association 9(2), e014373. 31928175 10.1161/JAHA.119.014373 PMC7033837

[r36] Galla S, Chakraborty S, Cheng X, Yeo J, Mell B, Zhang H, Mathew AV, Vijay-Kumar M and Joe B (2018) Disparate effects of antibiotics on hypertension. Physiological Genomics 50(10), 837–845. 30095376 10.1152/physiolgenomics.00073.2018 PMC6230872

[r37] Ge X, Zheng L, Zhuang R, Yu P, Xu Z, Liu G, Xi X, Zhou X and Fan H (2020) The gut microbial metabolite trimethylamine *N*-oxide and hypertension risk: A systematic review and dose-response meta-analysis. Advances in Nutrition 11(1), 66–76. 31269204 10.1093/advances/nmz064 PMC7442397

[r38] Golino HF, Amaral LSDB, Duarte SFP, Gomes CMA, Soares T J, Reis LA and Santos J (2014) Predicting increased blood pressure using machine learning. Journal of Obesity 2014, 637635. 24669313 10.1155/2014/637635 PMC3941962

[r39] Gomez-Arango LF, Barrett HL, McIntyre HD, Callaway LK, Morrison M and Dekker Nitert M (2016) Increased systolic and diastolic blood pressure is associated with altered gut microbiota composition and butyrate production in early pregnancy. Hypertension 68(4), 974–981. 27528065 10.1161/HYPERTENSIONAHA.116.07910

[r40] Gupta-Malhotra M, Banker A, Shete S, Hashmi SS, Tyson JE, Barratt MS, Hecht JT, Milewicz DM and Boerwinkle E (2015) Essential hypertension vs. secondary hypertension among children. American Journal of Hypertension 28(1), 73–80. 24842390 10.1093/ajh/hpu083 PMC4318949

[r41] Han M, Yang P, Zhong C and Ning K (2018) The human gut virome in hypertension. Frontiers in Microbiology 9, 3150. 30619215 10.3389/fmicb.2018.03150 PMC6305721

[r42] Hsu C-N, Hou C-Y, Chang-Chien G-P, Lin S and Tain Y-L (2020) Maternal *N*-acetylcysteine therapy prevents hypertension in spontaneously hypertensive rat offspring: Implications of hydrogen sulfide-generating pathway and gut microbiota. Antioxidants 9(9), 856. 32933169 10.3390/antiox9090856 PMC7554905

[r43] Hsu C-N, Hou C-Y, Lee C-T, Chan JYH and Tain Y-L (2019) The interplay between maternal and post-weaning high-fat diet and gut microbiota in the developmental programming of hypertension. Nutrients 11(9), 1982. 31443482 10.3390/nu11091982 PMC6769506

[r44] Hsu CN, Yu HR, Lin IC, Tiao MM, Huang LT, Hou CY, Chang-Chien GP, Lin S and Tain YL (2022) Sodium butyrate modulates blood pressure and gut microbiota in maternal tryptophan-free diet-induced hypertension rat offspring. Journal of Nutritional Biochemistry 108, 109090. 35724813 10.1016/j.jnutbio.2022.109090

[r45] Huart J, Leenders J, Taminiau B, Descy J, Saint-Remy A, Daube G, Krzesinski JM, Melin P, de Tullio P and Jouret F (2019) Gut microbiota and fecal levels of short-chain fatty acids differ upon 24-hour blood pressure levels in men. Hypertension 74(4), 1005–1013. 31352822 10.1161/HYPERTENSIONAHA.118.12588

[r46] Huć T, Nowinski A, Drapala A, Konopelski P and Ufnal M (2018) Indole and indoxyl sulfate, gut bacteria metabolites of tryptophan, change arterial blood pressure via peripheral and central mechanisms in rats. Pharmacological Research 130, 172–179. 29287686 10.1016/j.phrs.2017.12.025

[r47] International Consortium for Blood Pressure Genome-Wide Association Studies (2011) Genetic variants in novel pathways influence blood pressure and cardiovascular disease risk. Nature 478(7367), 103. 21909115 10.1038/nature10405 PMC3340926

[r48] Jackson MA, Verdi S, Maxan M-E, Shin CM, Zierer J, Bowyer RCE, Martin T, Williams FMK, Menni C, Bell JT, Spector TD and Steves CJ (2018) Gut microbiota associations with common diseases and prescription medications in a population-based cohort. Nature Communications 9(1), 1–8. 10.1038/s41467-018-05184-7 PMC6037668 29985401

[r49] Jama HA, Rhys-Jones D, Nakai M, Yao CK, Climie RE, Sata Y, Anderson D, Creek DJ, Head GA, Kaye DM, Mackay CR, Muir J and Marques FZ (2023) Prebiotic intervention with HAMSAB in untreated essential hypertensive patients assessed in a phase II randomized trial. Nature Cardiovascular Research 2, 35–43. 10.1038/s44161-022-00197-4 39196205

[r50] Jiang S, Shui Y, Cui Y, Tang C, Wang X, Qiu X, Hu W, Fei L, Li Y, Zhang S, Zhao L, Xu N, Dong F, Ren X, Liu R, Persson PB, Patzak A, Lai EY, Wei Q and Zheng Z (2021) Gut microbiota dependent trimethylamine *N*-oxide aggravates angiotensin II-induced hypertension. Redox Biology 46, 102115. 34474396 10.1016/j.redox.2021.102115 PMC8408632

[r51] Jie Z, Xia H, Zhong S-L, Feng Q, Li S, Liang S, Zhong H, Liu Z, Gao Y, Zhao H, Zhang D, Su Z, Fang Z, Lan Z, Li J, Xiao L, Li J, Li R, Li X, Li F, Ren H, Huang Y, Peng Y, Li G, Wen B, Dong B, Chen JY, Geng QS, Zhang ZW, Yang H, Wang J, Wang J, Zhang X, Madsen L, Brix S, Ning G, Xu X, Liu X, Hou Y, Jia H, He K and Kristiansen K (2017) The gut microbiome in atherosclerotic cardiovascular disease. Nature Communications 8(1), 845. 10.1038/s41467-017-00900-1 PMC5635030 29018189

[r52] Joe B, McCarthy CG, Edwards JM, Cheng X, Chakraborty S, Yang T, Golonka RM, Mell B, Yeo JY, Bearss NR, Furtado J, Saha P, Yeoh BS, Vijay-Kumar M and Wenceslau CF (2020) Microbiota introduced to germ-free rats restores vascular contractility and blood pressure. Hypertension 76(6), 1847–1855. 33070663 10.1161/HYPERTENSIONAHA.120.15939 PMC7666075

[r53] Joishy TK, Jha A, Oudah M, das S, Adak A, Deb D and Khan MR (2022) Human gut microbes associated with systolic blood pressure. International Journal of Hypertension 2022, 2923941. 35154822 10.1155/2022/2923941 PMC8831042

[r54] Kanai M, Akiyama M, Takahashi A, Matoba N, Momozawa Y, Ikeda M, Iwata N, Ikegawa S, Hirata M, Matsuda K, Kubo M, Okada Y and Kamatani Y (2018) Genetic analysis of quantitative traits in the Japanese population links cell types to complex human diseases. Nature Genetics 50(3), 390–400. 29403010 10.1038/s41588-018-0047-6

[r55] Kanegae H, Suzuki K, Fukatani K, Ito T, Harada N and Kario K (2020) Highly precise risk prediction model for new‐onset hypertension using artificial intelligence techniques. The Journal of Clinical Hypertension 22(3), 445–450. 31816148 10.1111/jch.13759 PMC8029685

[r56] Kaoutari AE, Armougom F, Gordon JI, Raoult D and Henrissat B (2013) The abundance and variety of carbohydrate-active enzymes in the human gut microbiota. Nature Reviews Microbiology 11(7), 497–504. 23748339 10.1038/nrmicro3050

[r57] Kim S, Goel R, Kumar A, Qi Y, Lobaton G, Hosaka K, Mohammed M, Handberg EM, Richards EM, Pepine CJ and Raizada MK (2018) Imbalance of gut microbiome and intestinal epithelial barrier dysfunction in patients with high blood pressure. Clinical Science 132(6), 701–718. 29507058 10.1042/CS20180087 PMC5955695

[r58] Koeth RA, Lam-Galvez BR, Kirsop J, Wang Z, Levison BS, Gu X, Copeland MF, Bartlett D, Cody DB, Dai HJ, Culley MK, Li XS, Fu X, Wu Y, Li L, DiDonato J, Tang WHW, Garcia-Garcia JC and Hazen SL (2019) L-carnitine in omnivorous diets induces an atherogenic gut microbial pathway in humans. The Journal of Clinical Investigation 129(1), 373–387. 30530985 10.1172/JCI94601 PMC6307959

[r59] Koeth RA, Wang Z, Levison BS, Buffa JA, Org E, Sheehy BT, Britt EB, Fu X, Wu Y, Li L, Smith JD, DiDonato JA, Chen J, Li H, Wu GD, Lewis JD, Warrier M, Brown JM, Krauss RM, Tang WHW, Bushman FD, Lusis AJ and Hazen SL (2013) Intestinal microbiota metabolism of L-carnitine, a nutrient in red meat, promotes atherosclerosis. Nature Medicine 19(5), 576–585. 10.1038/nm.3145 PMC3650111 23563705

[r60] Koh A, De Vadder F, Kovatcheva-Datchary P and Bäckhed F (2016) From dietary fiber to host physiology: Short-chain fatty acids as key bacterial metabolites. Cell 165(6), 1332–1345. 27259147 10.1016/j.cell.2016.05.041

[r61] Kurniansyah N, Goodman MO, Kelly TN, Elfassy T, Wiggins KL, Bis JC, Guo X, Palmas W, Taylor KD, Lin HJ, Haessler J, Gao Y, Shimbo D, Smith JA, Yu B, Feofanova EV, Smit RAJ, Wang Z, Hwang SJ, Liu S, Wassertheil-Smoller S, Manson JAE, Lloyd-Jones DM, Rich SS, Loos RJF, Redline S, Correa A, Kooperberg C, Fornage M, Kaplan RC, Psaty BM, Rotter JI, Arnett DK, Morrison AC, Franceschini N, Levy D, the NHLBI Trans-Omics in Precision Medicine (TOPMed) Consortium, Bis JC, Guo X, Taylor KD, Lin HJ, Haessler J, Gao Y, Smith JA, Liu S, Wassertheil-Smoller S, Manson JAE, Rich SS, Redline S, Correa A, Kooperberg C, Fornage M, Kaplan RC, Psaty BM, Rotter JI, Arnett DK, Franceschini N, Levy D, Sofer T and Sofer T (2022) A multi-ethnic polygenic risk score is associated with hypertension prevalence and progression throughout adulthood. Nature Communications 13(1), 1–13. 10.1038/s41467-022-31080-2 PMC9213527 35729114

[r62] Kyoung J, Atluri RR and Yang T (2022) Resistance to antihypertensive drugs: Is gut microbiota the missing link? Hypertension 79(10), 2138–2147. 35862173 10.1161/HYPERTENSIONAHA.122.19826

[r63] Kyoung J and Yang T (2022) Depletion of the gut microbiota enhances the blood pressure-lowering effect of captopril: Implication of the gut microbiota in resistant hypertension. Hypertension Research 45, 1505–1510. 35513487 10.1038/s41440-022-00921-4

[r64] Lacson RC, Baker B, Suresh H, Andriole K, Szolovits P and Lacson E (2019) Use of machine-learning algorithms to determine features of systolic blood pressure variability that predict poor outcomes in hypertensive patients. Clinical Kidney Journal 12(2), 206–212. 30976397 10.1093/ckj/sfy049 PMC6452173

[r65] Lewis S, Nash A, Li Q and Ahn T-H (2021) Comparison of 16S and whole genome dog microbiomes using machine learning. BioData Mining 14(1), 1–15. 34419136 10.1186/s13040-021-00270-x PMC8379800

[r66] Lewis CM and Vassos E (2020) Polygenic risk scores: From research tools to clinical instruments. Genome Medicine 12(1), 1–11. 10.1186/s13073-020-00742-5 PMC7236300 32423490

[r67] Li H, Liu B, Song J, An Z, Zeng X, Li J, Jiang J, Xie L and Wu W (2019b) Characteristics of gut microbiota in patients with hypertension and/or hyperlipidemia: A cross-sectional study on rural residents in Xinxiang County, Henan Province. Microorganisms 7(10), 399. 31561625 10.3390/microorganisms7100399 PMC6843550

[r68] Li C, Sun D, Liu J, Li M, Zhang B, Liu Y, Wang Z, Wen S and Zhou J (2019a) A prediction model of essential hypertension based on genetic and environmental risk factors in northern Han Chinese. International Journal of Medical Sciences 16(6), 793. 31337952 10.7150/ijms.33967 PMC6643104

[r69] Li HB, Xu ML, Xu XD, Tang YY, Jiang HL, Li L, Xia WJ, Cui N, Bai J, Dai ZM, Han B, Li Y, Peng B, Dong YY, Aryal S, Manandhar I, Eladawi MA, Shukla R, Kang YM, Joe B and Yang T (2022) *Faecalibacterium Prausnitzii* attenuates CKD via butyrate-renal GPR43 axis. Circulation Research 131, e120–e134. 36164984 10.1161/CIRCRESAHA.122.320184 PMC9588706

[r70] Li H-B, Yang T, Richards EM, Pepine CJ and Raizada MK (2020) Maternal treatment with captopril persistently alters gut-brain communication and attenuates hypertension of male offspring. Hypertension 75(5), 1315–1324. 32200676 10.1161/HYPERTENSIONAHA.120.14736 PMC7145738

[r71] Li J, Zhao F, Wang Y, Chen J, Tao J, Tian G, Wu S, Liu W, Cui Q, Geng B, Zhang W, Weldon R, Auguste K, Yang L, Liu X, Chen L, Yang X, Zhu B and Cai J (2017) Gut microbiota dysbiosis contributes to the development of hypertension. Microbiome 5(1), 1–19. 28143587 10.1186/s40168-016-0222-x PMC5286796

[r72] Liu J, An N, Ma C, Li X, Zhang J, Zhu W, Zhang Y and Li J (2018) Correlation analysis of intestinal flora with hypertension. Experimental and Therapeutic Medicine 16(3), 2325–2330. 30210587 10.3892/etm.2018.6500 PMC6122545

[r73] Liu Y, Jiang Q, Liu Z, Shen S, Ai J, Zhu Y and Zhou L (2021b) Alteration of gut microbiota relates to metabolic disorders in primary aldosteronism patients. Frontiers in Endocrinology 12, 667951. 34484110 10.3389/fendo.2021.667951 PMC8415980

[r74] Liu J-R, Miao H, Deng D-Q, Vaziri ND, Li P and Zhao Y-Y (2021a) Gut microbiota-derived tryptophan metabolism mediates renal fibrosis by aryl hydrocarbon receptor signaling activation. Cellular and Molecular Life Sciences 78(3), 909–922. 32965514 10.1007/s00018-020-03645-1 PMC11073292

[r75] Lloyd EE, Durgan DJ, Martini SR and Bryan RM (2015) Pathological effects of obstructive apneas during the sleep cycle in an animal model of cerebral small vessel disease. Hypertension 66(4), 913–917. 26259594 10.1161/HYPERTENSIONAHA.115.05764 PMC4567494

[r76] López-Martínez F, Núñez-Valdez ER, Crespo RG and García-Díaz V (2020) An artificial neural network approach for predicting hypertension using NHANES data. Scientific Reports 10(1), 1–14. 32606434 10.1038/s41598-020-67640-z PMC7327031

[r77] Louca P, Nogal A, Wells PM, Asnicar F, Wolf J, Steves CJ, Spector TD, Segata N, Berry SE, Valdes AM and Menni C (2021) Gut microbiome diversity and composition is associated with hypertension in women. Journal of Hypertension 39(9), 1810. 33973959 10.1097/HJH.0000000000002878 PMC7611529

[r78] Louca P, Tran TQB, Toit C, Christofidou P, Spector TD, Mangino M, Suhre K, Padmanabhan S and Menni C (2022) Machine learning integration of multimodal data identifies key features of blood pressure regulation. Ebiomedicine 84, 104243. 36084617 10.1016/j.ebiom.2022.104243 PMC9463529

[r79] Manolio TA, Collins FS, Cox NJ, Goldstein DB, Hindorff LA, Hunter DJ, McCarthy MI, Ramos EM, Cardon LR, Chakravarti A, Cho JH, Guttmacher AE, Kong A, Kruglyak L, Mardis E, Rotimi CN, Slatkin M, Valle D, Whittemore AS, Boehnke M, Clark AG, Eichler EE, Gibson G, Haines JL, Mackay TFC, McCarroll SA and Visscher PM (2009) Finding the missing heritability of complex diseases. Nature 461(7265), 747–753. 19812666 10.1038/nature08494 PMC2831613

[r80] Marques FZ, Mackay CR and Kaye DM (2018) Beyond gut feelings: How the gut microbiota regulates blood pressure. Nature Reviews Cardiology 15(1), 20–32. 28836619 10.1038/nrcardio.2017.120

[r81] Marques FZ, Nelson E, Chu P-Y, Horlock D, Fiedler A, Ziemann M, Tan JK, Kuruppu S, Rajapakse NW, el-Osta A, Mackay CR and Kaye DM (2017) High-fiber diet and acetate supplementation change the gut microbiota and prevent the development of hypertension and heart failure in hypertensive mice. Circulation 135(10), 964–977. 27927713 10.1161/CIRCULATIONAHA.116.024545

[r82] Martin AR, Kanai M, Kamatani Y, Okada Y, Neale BM and Daly MJ (2019) Clinical use of current polygenic risk scores may exacerbate health disparities. Nature Genetics 51(4), 584–591. 30926966 10.1038/s41588-019-0379-x PMC6563838

[r83] Mei X, Mell B, Cheng X, Yeo JY, Yang T, Chiu N and Joe B (2022) Beyond the gastrointestinal tract: Oral and sex-specific skin microbiota are associated with hypertension in rats with genetic disparities. Physiological Genomics 54, 242–250. 35503026 10.1152/physiolgenomics.00169.2021 PMC9208435

[r84] Mell B, Jala VR, Mathew AV, Byun J, Waghulde H, Zhang Y, Haribabu B, Vijay-Kumar M, Pennathur S and Joe B (2015) Evidence for a link between gut microbiota and hypertension in the dahl rat. Physiological Genomics 47(6), 187–197. 25829393 10.1152/physiolgenomics.00136.2014 PMC4451389

[r85] Minari J, Brothers KB and Morrison M (2018) Tensions in ethics and policy created by National Precision Medicine Programs. Human Genomics 12(1), 1–10. 29665847 10.1186/s40246-018-0151-9 PMC5904987

[r86] Munukka E, Wiklund P, Pekkala S, Völgyi E, Xu L, Cheng S, Lyytikäinen A, Marjomäki V, Alen M, Vaahtovuo J, Keinänen-Kiukaanniemi S and Cheng S (2012) Women with and without metabolic disorder differ in their gut microbiota composition. Obesity 20(5), 1082–1087. 22293842 10.1038/oby.2012.8

[r87] Mushtaq N, Hussain S, Zhang S, Yuan L, Li H, Ullah S, Wang Y and Xu J (2019) Molecular characterization of alterations in the intestinal microbiota of patients with grade 3 hypertension. International Journal of Molecular Medicine 44(2), 513–522. 31173179 10.3892/ijmm.2019.4235 PMC6605625

[r88] Nakai M, Ribeiro RV, Stevens BR, Gill P, Muralitharan RR, Yiallourou S, Muir J, Carrington M, Head GA, Kaye DM and Marques FZ (2021) Essential hypertension is associated with changes in gut microbial metabolic pathways: A multisite analysis of ambulatory blood pressure. Hypertension 78(3), 804–815. 34333988 10.1161/HYPERTENSIONAHA.121.17288

[r89] Natividad JM, Agus A, Planchais J, Lamas B, Jarry AC, Martin R, Michel ML, Chong-Nguyen C, Roussel R, Straube M, Jegou S, McQuitty C, le Gall M, da Costa G, Lecornet E, Michaudel C, Modoux M, Glodt J, Bridonneau C, Sovran B, Dupraz L, Bado A, Richard ML, Langella P, Hansel B, Launay JM, Xavier RJ, Duboc H and Sokol H (2018) Impaired aryl hydrocarbon receptor ligand production by the gut microbiota is a key factor in metabolic syndrome. Cell Metabolism 28(5), 737–749. 30057068 10.1016/j.cmet.2018.07.001

[r90] Nelson JW, Phillips SC, Ganesh BP, Petrosino JF, Durgan DJ and Bryan RM (2021) The gut microbiome contributes to blood‐brain barrier disruption in spontaneously hypertensive stroke prone rats. The FASEB Journal 35(2), e21201. 33496989 10.1096/fj.202001117R PMC8238036

[r91] Olczak KJ, Taylor‐Bateman V, Nicholls HL, Traylor M, Cabrera CP and Munroe PB (2021) Hypertension genetics past, present and future applications. Journal of Internal Medicine 290(6), 1130–1152. 34166551 10.1111/joim.13352

[r92] Overby HB and Ferguson JF (2021) Gut microbiota-derived short-chain fatty acids facilitate microbiota: Host cross talk and modulate obesity and hypertension. Current Hypertension Reports 23(2), 1–10. 10.1007/s11906-020-01125-2 PMC7992370 33537923

[r93] Padmanabhan S and Dominiczak AF (2021) Genomics of hypertension: The road to precision medicine. Nature Reviews Cardiology 18(4), 235–250. 33219353 10.1038/s41569-020-00466-4

[r94] Padmanabhan S and Joe B (2017) Towards precision medicine for hypertension: A review of genomic, epigenomic, and microbiomic effects on blood pressure in experimental rat models and humans. Physiological Reviews 97(4), 1469–1528. 28931564 10.1152/physrev.00035.2016 PMC6347103

[r95] Padmanabhan S, Tran TQB and Dominiczak AF (2021) Artificial intelligence in hypertension: Seeing through a glass darkly. Circulation Research 128(7), 1100–1118. 33793339 10.1161/CIRCRESAHA.121.318106

[r96] Palmu J, Salosensaari A, Havulinna AS, Cheng S, Inouye M, Jain M, Salido RA, Sanders K, Brennan C, Humphrey GC, Sanders JG, Vartiainen E, Laatikainen T, Jousilahti P, Salomaa V, Knight R, Lahti L and Niiranen TJ (2020) Association between the gut microbiota and blood pressure in a population cohort of 6953 individuals. Journal of the American Heart Association 9(15), e016641. 32691653 10.1161/JAHA.120.016641 PMC7792269

[r97] Pan F, He P, Chen F, Pu X, Zhao Q and Zheng D (2019) Deep learning-based automatic blood pressure measurement: Evaluation of the effect of deep breathing, talking and arm movement. Annals of Medicine 51(7–8), 397–403. 31724891 10.1080/07853890.2019.1694170 PMC7877882

[r98] Parcha V, Pampana A, Bress AP, Irvin MR, Arora G and Arora P (2022) Association of Polygenic Risk Score with blood pressure and adverse cardiovascular outcomes in individuals with type II diabetes: Insights from the ACCORD trial. Hypertension 79(5), e100–e102. 35369713 10.1161/HYPERTENSIONAHA.122.18976 PMC9010352

[r99] Paré G, Mao S and Deng WQ (2017) A machine-learning heuristic to improve gene score prediction of polygenic traits. Scientific Reports 7(1), 1–11. 28979001 10.1038/s41598-017-13056-1 PMC5627249

[r100] Persell SD, Peprah YA, Lipiszko D, Lee JY, Li JJ, Ciolino JD, Karmali KN and Sato H (2020) Effect of home blood pressure monitoring via a smartphone hypertension coaching application or tracking application on adults with uncontrolled hypertension: A randomized clinical trial. JAMA Network Open 3(3), e200255. 32119093 10.1001/jamanetworkopen.2020.0255 PMC7052730

[r101] Queipo-Ortuño MI, Boto-Ordóñez M, Murri M, Gomez-Zumaquero JM, Clemente-Postigo M, Estruch R, Cardona Diaz F, Andrés-Lacueva C and Tinahones FJ (2012) Influence of red wine polyphenols and ethanol on the gut microbiota ecology and biochemical biomarkers. The American Journal of Clinical Nutrition 95(6), 1323–1334. 22552027 10.3945/ajcn.111.027847

[r102] Quintanilha JCF, Etheridge AS, Graynor BJ, Larson NB, Crona DJ, Mitchell BD and Innocenti F (2022) Polygenic risk scores for blood pressure to assess the risk of severe bevacizumab‐induced hypertension in cancer patients (Alliance). Clinical Pharmacology & Therapeutics 112, 364–371. 35527502 10.1002/cpt.2635 PMC9296545

[r103] Ried K, Travica N and Sali A (2018) The effect of Kyolic aged garlic extract on gut microbiota, inflammation, and cardiovascular markers in hypertensives: The GarGIC trial. Frontiers in Nutrition 5, 122. 30619868 10.3389/fnut.2018.00122 PMC6297383

[r104] Robles-Vera I, de la Visitación N, Sánchez M, Gómez-Guzmán M, Jiménez R, Moleón J, González-Correa C, Romero M, Yang T, Raizada MK, Toral M and Duarte J (2020) Mycophenolate improves brain–gut Axis inducing remodeling of gut microbiota in DOCA-salt hypertensive rats. Antioxidants 9(12), 1199. 33260593 10.3390/antiox9121199 PMC7761232

[r105] Robles-Vera I, Toral M, la Visitación N, Sánchez M, Gómez-Guzmán M, Romero M, Yang T, Izquierdo-Garcia JL, Jiménez R, Ruiz-Cabello J, Guerra-Hernández E, Raizada MK, Pérez-Vizcaíno F and Duarte J (2020c) Probiotics prevent dysbiosis and the rise in blood pressure in genetic hypertension: Role of short‐chain fatty acids. Molecular Nutrition & Food Research 64(6), 1900616. 10.1002/mnfr.201900616 31953983

[r106] Robles-Vera I, Toral M, Visitación N, Sánchez M, Gómez-Guzmán M, Muñoz R, Algieri F, Vezza T, Jiménez R, Gálvez J, Romero M, Redondo JM and Duarte J (2020b) Changes to the gut microbiota induced by losartan contributes to its antihypertensive effects. British Journal of Pharmacology 177(9), 2006–2023. 31883108 10.1111/bph.14965 PMC7161554

[r107] Robles-Vera I, Toral M, de la Visitación N, Sánchez M, Romero M, Olivares M, Jiménez R and Duarte J (2018) The probiotic lactobacillus fermentum prevents dysbiosis and vascular oxidative stress in rats with hypertension induced by chronic nitric oxide blockade. Molecular Nutrition & Food Research 62(19), 1800298. 10.1002/mnfr.201800298 30028078

[r108] Robles-Vera I, Visitación N, Toral M, Sánchez M, Romero M, Gómez-Guzmán M, Yang T, Izquierdo-García JL, Guerra-Hernández E, Ruiz-Cabello J, Raizada MK, Pérez-Vizcaíno F, Jiménez R and Duarte J (2020a) Probiotic Bifidobacterium breve prevents DOCA‐salt hypertension. The FASEB Journal 34(10), 13626–13640. 32780919 10.1096/fj.202001532R

[r109] Santisteban MM, Qi Y, Zubcevic J, Kim S, Yang T, Shenoy V, Cole-Jeffrey CT, Lobaton GO, Stewart DC, Rubiano A, Simmons CS, Garcia-Pereira F, Johnson RD, Pepine CJ and Raizada MK (2017) Hypertension-linked pathophysiological alterations in the gut. Circulation Research 120(2), 312–323. 27799253 10.1161/CIRCRESAHA.116.309006 PMC5250568

[r110] Sapkota Y, Li N, Pierzynski J, Mulrooney DA, Ness KK, Morton LM, Michael JR, Zhang J, Bhatia S, Armstrong GT, Hudson MM, Robison LL and Yasui Y (2021) Contribution of polygenic risk to hypertension among long-term survivors of childhood cancer. Cardio Oncology 3(1), 76–84. 10.1016/j.jaccao.2021.01.007 PMC8026142 33842896

[r111] Sato N, Fudono A, Imai C, Takimoto H, Tarui I, Aoyama T, Yago S, Okamitsu M, Mizutani S and Miyasaka N (2021) Placenta mediates the effect of maternal hypertension polygenic score on offspring birth weight: A study of birth cohort with fetal growth velocity data. BMC Medicine 19(1), 1–13. 34732167 10.1186/s12916-021-02131-0 PMC8567693

[r112] Schrumpf F, Frenzel P, Aust C, Osterhoff G and Fuchs M (2021) Assessment of non-invasive blood pressure prediction from ppg and rppg signals using deep learning. Sensors 21(18), 6022. 34577227 10.3390/s21186022 PMC8472879

[r113] Shah RD, Tang Z-Z, Chen G, Huang S and Ferguson JF (2020) Soy food intake associates with changes in the metabolome and reduced blood pressure in a gut microbiota dependent manner. Nutrition, Metabolism and Cardiovascular Diseases 30(9), 1500–1511. 10.1016/j.numecd.2020.05.001 PMC7483644 32620337

[r114] Sharma RK, Yang T, Oliveira AC, Lobaton GO, Aquino V, Kim S, Richards EM, Pepine CJ, Sumners C and Raizada MK (2019) Microglial cells impact gut microbiota and gut pathology in angiotensin II-induced hypertension. Circulation Research 124(5), 727–736. 30612527 10.1161/CIRCRESAHA.118.313882 PMC6395495

[r115] Sherman SB, Sarsour N, Salehi M, Schroering A, Mell B, Joe B and Hill JW (2018) Prenatal androgen exposure causes hypertension and gut microbiota dysbiosis. Gut Microbes 9(5), 400–421. 29469650 10.1080/19490976.2018.1441664 PMC6219642

[r116] Shi H, Nelson JW, Phillips S, Petrosino JF, Bryan RM and Durgan DJ (2022) Alterations of the gut microbial community structure and function with aging in the spontaneously hypertensive stroke prone rat. Scientific Reports 12(1), 1–9. 35595870 10.1038/s41598-022-12578-7 PMC9122926

[r117] Shi F, Shi H, Phillips S, Zhang B, Ayyaswamy S, Bryan R and Durgan D (2021a) Examining the role of extacellular vesicles in blood pressure regulation. The FASEB Journal 35(S1).

[r118] Shi H, Zhang B, Abo-Hamzy T, Nelson JW, Ambati CSR, Petrosino JF, Bryan Jr RM and Durgan DJ (2021b) Restructuring the gut microbiota by intermittent fasting lowers blood pressure. Circulation Research 128(9), 1240–1254. 33596669 10.1161/CIRCRESAHA.120.318155 PMC8085162

[r119] Silveira-Nunes G, Durso DF Jr, LRAO, Cunha EHM, Maioli TU, Vieira AT, Speziali E, Corrêa-Oliveira R, Martins-Filho OA, Teixeira-Carvalho A, Franceschi C, Rampelli S, Turroni S, Brigidi P and Faria AMC (2020) Hypertension is associated with intestinal microbiota dysbiosis and inflammation in a Brazilian population. Frontiers in Pharmacology 11, 258. 32226382 10.3389/fphar.2020.00258 PMC7080704

[r120] Soh DCK, Ng EYK, Jahmunah V, Oh SL, San TR and Acharya UR (2020) A computational intelligence tool for the detection of hypertension using empirical mode decomposition. Computers in Biology and Medicine 118, 103630. 32174317 10.1016/j.compbiomed.2020.103630

[r121] Steinthorsdottir V, McGinnis R, Williams NO, Stefansdottir L, Thorleifsson G, Shooter S, Fadista J, Sigurdsson JK, Auro KM, Berezina G, Borges MC, Bumpstead S, Bybjerg-Grauholm J, Colgiu I, Dolby VA, Dudbridge F, Engel SM, Franklin CS, Frigge ML, Frisbaek Y, Geirsson RT, Geller F, Gretarsdottir S, Gudbjartsson DF, Harmon Q, Hougaard DM, Hegay T, Helgadottir A, Hjartardottir S, Jääskeläinen T, Johannsdottir H, Jonsdottir I, Juliusdottir T, Kalsheker N, Kasimov A, Kemp JP, Kivinen K, Klungsøyr K, Lee WK, Melbye M, Miedzybrodska Z, Moffett A, Najmutdinova D, Nishanova F, Olafsdottir T, Perola M, Pipkin FB, Poston L, Prescott G, Saevarsdottir S, Salimbayeva D, Scaife PJ, Skotte L, Staines-Urias E, Stefansson OA, Sørensen KM, Thomsen LCV, Tragante V, Trogstad L, Simpson NAB; FINNPEC Consortium; GOPEC Consortium; Aripova T, Casas JP, Dominiczak AF, Walker JJ, Thorsteinsdottir U, Iversen AC, Feenstra B, Lawlor DA, Boyd HA, Magnus P, Laivuori H, Zakhidova N, Svyatova G, Stefansson K and Morgan L (2020) Genetic predisposition to hypertension is associated with preeclampsia in European and central Asian women. Nature Communications 11(1), 1–14. 10.1038/s41467-020-19733-6 PMC7688949 33239696

[r122] Stilp AM, Emery LS, Broome JG, Buth EJ, Khan AT, Laurie CA, Wang FF, Wong Q, Chen D, D’Augustine CM, Heard-Costa NL, Hohensee CR, Johnson WC, Juarez LD, Liu J, Mutalik KM, Raffield LM, Wiggins KL, de Vries PS, Kelly TN, Kooperberg C, Natarajan P, Peloso GM, Peyser PA, Reiner AP, Arnett DK, Aslibekyan S, Barnes KC, Bielak LF, Bis JC, Cade BE, Chen MH, Correa A, Cupples LA, de Andrade M, Ellinor PT, Fornage M, Franceschini N, Gan W, Ganesh SK, Graffelman J, Grove ML, Guo X, Hawley NL, Hsu WL, Jackson RD, Jaquish CE, Johnson AD, Kardia SLR, Kelly S, Lee J, Mathias RA, McGarvey ST, Mitchell BD, Montasser ME, Morrison AC, North KE, Nouraie SM, Oelsner EC, Pankratz N, Rich SS, Rotter JI, Smith JA, Taylor KD, Vasan RS, Weeks DE, Weiss ST, Wilson CG, Yanek LR, Psaty BM, Heckbert SR and Laurie CC (2021) A system for phenotype harmonization in the national heart, lung, and blood institute trans-omics for precision medicine (TOPMed) program. American Journal of Epidemiology 190(10), 1977–1992. 33861317 10.1093/aje/kwab115 PMC8485147

[r123] Sugrue LP and Desikan RS (2019) What are polygenic scores and why are they important? JAMA 321(18), 1820–1821. 30958510 10.1001/jama.2019.3893

[r124] Sun S, Lulla A, Sioda M, Winglee K, Wu MC, Jacobs Jr DR, Shikany JM, Lloyd-Jones DM, Launer LJ, Fodor AA and Meyer KA (2019) Gut microbiota composition and blood pressure: The CARDIA study. Hypertension 73(5), 998–1006. 30905192 10.1161/HYPERTENSIONAHA.118.12109 PMC6458072

[r125] Sung YJ, Winkler TW, de Las Fuentes L, Bentley AR, Brown MR, Kraja AT, Schwander K, Ntalla I, Guo X, Franceschini N, Lu Y, Cheng CY, Sim X, Vojinovic D, Marten J, Musani SK, Li C, Feitosa MF, Kilpeläinen TO, Richard MA, Noordam R, Aslibekyan S, Aschard H, Bartz TM, Dorajoo R, Liu Y, Manning AK, Rankinen T, Smith AV, Tajuddin SM, Tayo BO, Warren HR, Zhao W, Zhou Y, Matoba N, Sofer T, Alver M, Amini M, Boissel M, Chai JF, Chen X, Divers J, Gandin I, Gao C, Giulianini F, Goel A, Harris SE, Hartwig FP, Horimoto ARVR, Hsu FC, Jackson AU, Kähönen M, Kasturiratne A, Kühnel B, Leander K, Lee WJ, Lin KH, ’an Luan J, McKenzie C, Meian H, Nelson CP, Rauramaa R, Schupf N, Scott RA, Sheu WHH, Stančáková A, Takeuchi F, van der Most P, Varga TV, Wang H, Wang Y, Ware EB, Weiss S, Wen W, Yanek LR, Zhang W, Zhao JH, Afaq S, Alfred T, Amin N, Arking D, Aung T, Barr RG, Bielak LF, Boerwinkle E, Bottinger EP, Braund PS, Brody JA, Broeckel U, Cabrera CP, Cade B, Caizheng Y, Campbell A, Canouil M, Chakravarti A, CHARGE Neurology Working Group, Chauhan G, Christensen K, Cocca M, COGENT-Kidney Consortium, Collins FS, Connell JM, de Mutsert R, de Silva HJ, Debette S, Dörr M, Duan Q, Eaton CB, Ehret G, Evangelou E, Faul JD, Fisher VA, Forouhi NG, Franco OH, Friedlander Y, Gao H, GIANT Consortium, Gigante B, Graff M, Gu CC, Gu D, Gupta P, Hagenaars SP, Harris TB, He J, Heikkinen S, Heng CK, Hirata M, Hofman A, Howard BV, Hunt S, Irvin MR, Jia Y, Joehanes R, Justice AE, Katsuya T, Kaufman J, Kerrison ND, Khor CC, Koh WP, Koistinen HA, Komulainen P, Kooperberg C, Krieger JE, Kubo M, Kuusisto J, Langefeld CD, Langenberg C, Launer LJ, Lehne B, Lewis CE, Li Y, Lifelines Cohort Study, Lim SH, Lin S, Liu CT, Liu J, Liu J, Liu K, Liu Y, Loh M, Lohman KK, Long J, Louie T, Mägi R, Mahajan A, Meitinger T, Metspalu A, Milani L, Momozawa Y, Morris AP, Mosley TH Jr, Munson P, Murray AD, Nalls MA, Nasri U, Norris JM, North K, Ogunniyi A, Padmanabhan S, Palmas WR, Palmer ND, Pankow JS, Pedersen NL, Peters A, Peyser PA, Polasek O, Raitakari OT, Renström F, Rice TK, Ridker PM, Robino A, Robinson JG, Rose LM, Rudan I, Sabanayagam C, Salako BL, Sandow K, Schmidt CO, Schreiner PJ, Scott WR, Seshadri S, Sever P, Sitlani CM, Smith JA, Snieder H, Starr JM, Strauch K, Tang H, Taylor KD, Teo YY, Tham YC, Uitterlinden AG, Waldenberger M, Wang L, Wang YX, Wei WB, Williams C, Wilson G, Wojczynski MK, Yao J, Yuan JM, Zonderman AB, Becker DM, Boehnke M, Bowden DW, Chambers JC, Chen YI, de Faire U, Deary IJ, Esko T, Farrall M, Forrester T, Franks PW, Freedman BI, Froguel P, Gasparini P, Gieger C, Horta BL, Hung YJ, Jonas JB, Kato N, Kooner JS, Laakso M, Lehtimäki T, Liang KW, Magnusson PKE, Newman AB, Oldehinkel AJ, Pereira AC, Redline S, Rettig R, Samani NJ, Scott J, Shu XO, van der Harst P, Wagenknecht LE, Wareham NJ, Watkins H, Weir DR, Wickremasinghe AR, Wu T, Zheng W, Kamatani Y, Laurie CC, Bouchard C, Cooper RS, Evans MK, Gudnason V, Kardia SLR, Kritchevsky SB, Levy D, O’Connell JR, Psaty BM, van Dam R, Sims M, Arnett DK, Mook-Kanamori DO, Kelly TN, Fox ER, Hayward C, Fornage M, Rotimi CN, Province MA, van Duijn C, Tai ES, Wong TY, Loos RJF, Reiner AP, Rotter JI, Zhu X, Bierut LJ, Gauderman WJ, Caulfield MJ, Elliott P, Rice K, Munroe PB, Morrison AC, Cupples LA, Rao DC and Chasman DI (2018) A large-scale multi-ancestry genome-wide study accounting for smoking behavior identifies multiple significant loci for blood pressure. The American Journal of Human Genetics 102(3), 375–400. 29455858 10.1016/j.ajhg.2018.01.015 PMC5985266

[r126] Surendran P, Feofanova EV, Lahrouchi N, Ntalla I, Karthikeyan S, Cook J, Chen L, Mifsud B, Yao C, Kraja AT, Cartwright JH, Hellwege JN, Giri A, Tragante V, Thorleifsson G, Liu DJ, Prins BP, Stewart ID, Cabrera CP, Eales JM, Akbarov A, Auer PL, Bielak LF, Bis JC, Braithwaite VS, Brody JA, Daw EW, Warren HR, Drenos F, Nielsen SF, Faul JD, Fauman EB, Fava C, Ferreira T, Foley CN, Franceschini N, Gao H, Giannakopoulou O, Giulianini F, Gudbjartsson DF, Guo X, Harris SE, Havulinna AS, Helgadottir A, Huffman JE, Hwang SJ, Kanoni S, Kontto J, Larson MG, Li-Gao R, Lindström J, Lotta LA, Lu Y, Luan J’, Mahajan A, Malerba G, Masca NGD, Mei H, Menni C, Mook-Kanamori DO, Mosen-Ansorena D, Müller-Nurasyid M, Paré G, Paul DS, Perola M, Poveda A, Rauramaa R, Richard M, Richardson TG, Sepúlveda N, Sim X, Smith AV, Smith JA, Staley JR, Stanáková A, Sulem P, Thériault S, Thorsteinsdottir U, Trompet S, Varga TV, Velez Edwards DR, Veronesi G, Weiss S, Willems SM, Yao J, Young R, Yu B, Zhang W, Zhao JH, Zhao W, Zhao W, Evangelou E, Aeschbacher S, Asllanaj E, Blankenberg S, Bonnycastle LL, Bork-Jensen J, Brandslund I, Braund PS, Burgess S, Cho K, Christensen C, Connell J, Mutsert R, Dominiczak AF, Dörr M, Eiriksdottir G, Farmaki AE, Gaziano JM, Grarup N, Grove ML, Hallmans G, Hansen T, Have CT, Heiss G, Jørgensen ME, Jousilahti P, Kajantie E, Kamat M, Käräjämäki AM, Karpe F, Koistinen HA, Kovesdy CP, Kuulasmaa K, Laatikainen T, Lannfelt L, Lee IT, Lee WJ, LifeLines Cohort Study, de Boer RA, van der Harst P, van der Meer P, Verweij N, Linneberg A, Martin LW, Moitry M, Nadkarni G, Neville MJ, Palmer CNA, Papanicolaou GJ, Pedersen O, Peters J, Poulter N, Rasheed A, Rasmussen KL, Rayner NW, Mägi R, Renström F, Rettig R, Rossouw J, Schreiner PJ, Sever PS, Sigurdsson EL, Skaaby T, Sun YV, Sundstrom J, Thorgeirsson G, Esko T, Trabetti E, Tsao PS, Tuomi T, Turner ST, Tzoulaki I, Vaartjes I, Vergnaud AC, Willer CJ, Wilson PWF, Witte DR, Yonova-Doing E, Zhang H, Aliya N, Almgren P, Amouyel P, Asselbergs FW, Barnes MR, Blakemore AI, Boehnke M, Bots ML, Bottinger EP, Buring JE, Chambers JC, Chen YDI, Chowdhury R, Conen D, Correa A, Davey Smith G, Boer RA, Deary IJ, Dedoussis G, Deloukas P, di Angelantonio E, Elliott P, EPIC-CVD, Butterworth AS, Danesh J, EPIC-InterAct, Langenberg C, Deloukas P, McCarthy MI, Franks PW, Rolandsson O, Wareham NJ, Felix SB, Ferrières J, Ford I, Fornage M, Franks PW, Franks S, Frossard P, Gambaro G, Gaunt TR, Groop L, Gudnason V, Harris TB, Hayward C, Hennig BJ, Herzig KH, Ingelsson E, Tuomilehto J, Järvelin MR, Jukema JW, Kardia SLR, Kee F, Kooner JS, Kooperberg C, Launer LJ, Lind L, Loos RJF, Majumder A S, Laakso M, McCarthy MI, Melander O, Mohlke KL, Murray AD, Nordestgaard BG, Orho-Melander M, Packard CJ, Padmanabhan S, Palmas W, Polasek O, Porteous DJ, Prentice AM, Province MA, Relton CL, Rice K, Ridker PM, Rolandsson O, Rosendaal FR, Rotter JI, Rudan I, Salomaa V, Samani NJ, Sattar N, Sheu WHH, Smith BH, Soranzo N, Spector TD, Starr JM, Sebert S, Taylor KD, Lakka TA, Timpson NJ, Tobin MD, Understanding Society Scientific Group, Prins BP, Zeggini E, van der Harst P, van der Meer P, Ramachandran VS, Verweij N, Virtamo J, Völker U, Weir DR, Zeggini E, Charchar FJ, Million Veteran Program, Hellwege JN, Giri A, Edwards DRV, Cho K, Gaziano JM, Kovesdy CP, Sun YV, Tsao PS, Wilson PWF, Edwards TL, Hung AM, O’Donnell CJ, Wareham NJ, Langenberg C, Tomaszewski M, Butterworth AS, Caulfield MJ, Danesh J, Edwards TL, Holm H, Hung AM, Lindgren CM, Liu C, Manning AK, Morris AP, Morrison AC, O’Donnell CJ, Psaty BM, Saleheen D, Stefansson K, Boerwinkle E, Chasman DI, Levy D, Newton-Cheh C, Munroe PB and Howson JMM (2020) Discovery of rare variants associated with blood pressure regulation through meta-analysis of 1.3 million individuals. Nature Genetics 52(12), 1314–1332. 33230300 10.1038/s41588-020-00713-x PMC7610439

[r127] Tain YL, Lee WC, Wu KLH, Leu S and Chan JYH (2018) Resveratrol prevents the development of hypertension programmed by maternal plus post-weaning high-fructose consumption through modulation of oxidative stress, nutrient-sensing signals, and gut microbiota. Molecular Nutrition and Food Research 62(15), 1800066. 10.1002/mnfr.201800066 29710384

[r128] Takagi T, Naito Y, Kashiwagi S, Uchiyama K, Mizushima K, Kamada K, Ishikawa T, Inoue R, Okuda K, Tsujimoto Y, Ohnogi H and Itoh Y (2020) Changes in the gut microbiota are associated with hypertension, hyperlipidemia, and type 2 diabetes mellitus in Japanese subjects. Nutrients 12(10), 2996. 33007825 10.3390/nu12102996 PMC7601322

[r129] Taliun D, Harris DN, Kessler MD, Carlson J, Szpiech ZA, Torres R, Taliun SAG, Corvelo A, Gogarten SM, Kang HM, Pitsillides AN, LeFaive J, Lee SB, Tian X, Browning BL, das S, Emde AK, Clarke WE, Loesch DP, Shetty AC, Blackwell TW, Smith AV, Wong Q, Liu X, Conomos MP, Bobo DM, Aguet F, Albert C, Alonso A, Ardlie KG, Arking DE, Aslibekyan S, Auer PL, Barnard J, Barr RG, Barwick L, Becker LC, Beer RL, Benjamin EJ, Bielak LF, Blangero J, Boehnke M, Bowden DW, Brody JA, Burchard EG, Cade BE, Casella JF, Chalazan B, Chasman DI, Chen YDI, Cho MH, Choi SH, Chung MK, Clish CB, Correa A, Curran JE, Custer B, Darbar D, Daya M, de Andrade M, DeMeo DL, Dutcher SK, Ellinor PT, Emery LS, Eng C, Fatkin D, Fingerlin T, Forer L, Fornage M, Franceschini N, Fuchsberger C, Fullerton SM, Germer S, Gladwin MT, Gottlieb DJ, Guo X, Hall ME, He J, Heard-Costa NL, Heckbert SR, Irvin MR, Johnsen JM, Johnson AD, Kaplan R, Kardia SLR, Kelly T, Kelly S, Kenny EE, Kiel DP, Klemmer R, Konkle BA, Kooperberg C, Köttgen A, Lange LA, Lasky-Su J, Levy D, Lin X, Lin KH, Liu C, Loos RJF, Garman L, Gerszten R, Lubitz SA, Lunetta KL, Mak ACY, Manichaikul A, Manning AK, Mathias RA, McManus DD, McGarvey ST, Meigs JB, Meyers DA, Mikulla JL, Minear MA, Mitchell BD, Mohanty S, Montasser ME, Montgomery C, Morrison AC, Murabito JM, Natale A, Natarajan P, Nelson SC, North KE, O’Connell JR, Palmer ND, Pankratz N, Peloso GM, Peyser PA, Pleiness J, Post WS, Psaty BM, Rao DC, Redline S, Reiner AP, Roden D, Rotter JI, Ruczinski I, Sarnowski C, Schoenherr S, Schwartz DA, Seo JS, Seshadri S, Sheehan VA, Sheu WH, Shoemaker MB, Smith NL, Smith JA, Sotoodehnia N, Stilp AM, Tang W, Taylor KD, Telen M, Thornton TA, Tracy RP, van den Berg DJ, Vasan RS, Viaud-Martinez KA, Vrieze S, Weeks DE, Weir BS, Weiss ST, Weng LC, Willer CJ, Zhang Y, Zhao X, Arnett DK, Ashley-Koch AE, Barnes KC, Boerwinkle E, Gabriel S, Gibbs R, Rice KM, Rich SS, Silverman EK, Qasba P, Gan W, NHLBI Trans-Omics for Precision Medicine (TOPMed) Consortium, Abe N, Almasy L, Ament S, Anderson P, Anugu P, Applebaum-Bowden D, Assimes T, Avramopoulos D, Barron-Casella E, Beaty T, Beck G, Becker D, Beitelshees A, Benos T, Bezerra M, Bis J, Bowler R, Broeckel U, Broome J, Bunting K, Bustamante C, Buth E, Cardwell J, Carey V, Carty C, Casaburi R, Castaldi P, Chaffin M, Chang C, Chang YC, Chavan S, Chen BJ, Chen WM, Chuang LM, Chung RH, Comhair S, Cornell E, Crandall C, Crapo J, Curtis J, Damcott C, David S, Davis C, Fuentes L, DeBaun M, Deka R, Devine S, Duan Q, Duggirala R, Durda JP, Eaton C, Ekunwe L, el Boueiz A, Erzurum S, Farber C, Flickinger M, Fornage M, Frazar C, Fu M, Fulton L, Gao S, Gao Y, Gass M, Gelb B, Geng XP, Geraci M, Ghosh A, Gignoux C, Glahn D, Gong DW, Goring H, Graw S, Grine D, Gu CC, Guan Y, Gupta N, Haessler J, Hawley NL, Heavner B, Herrington D, Hersh C, Hidalgo B, Hixson J, Hobbs B, Hokanson J, Hong E, Hoth K, Hsiung CA, Hung YJ, Huston H, Hwu CM, Jackson R, Jain D, Jhun MA, Johnson C, Johnston R, Jones K, Kathiresan S, Khan A, Kim W, Kinney G, Kramer H, Lange C, Lange E, Lange L, Laurie C, LeBoff M, Lee J, Lee SS, Lee WJ, Levine D, Lewis J, Li X, Li Y, Lin H, Lin H, Lin KH, Liu S, Liu Y, Liu Y, Luo J, Mahaney M, Make B, Manson JA, Margolin L, Martin L, Mathai S, May S, McArdle P, McDonald ML, McFarland S, McGoldrick D, McHugh C, Mei H, Mestroni L, Min N, Minster RL, Moll M, Moscati A, Musani S, Mwasongwe S, Mychaleckyj JC, Nadkarni G, Naik R, Naseri T, Nekhai S, Neltner B, Ochs-Balcom H, Paik D, Pankow J, Parsa A, Peralta JM, Perez M, Perry J, Peters U, Phillips LS, Pollin T, Becker JP, Boorgula MP, Preuss M, Qiao D, Qin Z, Rafaels N, Raffield L, Rasmussen-Torvik L, Ratan A, Reed R, Regan E, Reupena M‘S, Roselli C, Russell P, Ruuska S, Ryan K, Sabino EC, Saleheen D, Salimi S, Salzberg S, Sandow K, Sankaran VG, Scheller C, Schmidt E, Schwander K, Sciurba F, Seidman C, Seidman J, Sherman SL, Shetty A, Sheu WHH, Silver B, Smith J, Smith T, Smoller S, Snively B, Snyder M, Sofer T, Storm G, Streeten E, Sung YJ, Sylvia J, Szpiro A, Sztalryd C, Tang H, Taub M, Taylor M, Taylor S, Threlkeld M, Tinker L, Tirschwell D, Tishkoff S, Tiwari H, Tong C, Tsai M, Vaidya D, VandeHaar P, Walker T, Wallace R, Walts A, Wang FF, Wang H, Watson K, Wessel J, Williams K, Williams LK, Wilson C, Wu J, Xu H, Yanek L, Yang I, Yang R, Zaghloul N, Zekavat M, Zhao SX, Zhao W, Zhi D, Zhou X, Zhu X, Papanicolaou GJ, Nickerson DA, Browning SR, Zody MC, Zöllner S, Wilson JG, Cupples LA, Laurie CC, Jaquish CE, Hernandez RD, O’Connor TD and Abecasis GR (2021) Sequencing of 53,831 diverse genomes from the NHLBI TOPMed program. Nature 590(7845), 290–299. 33568819 10.1038/s41586-021-03205-y PMC7875770

[r130] The Million Veteran Program, Evangelou E, Warren HR, Mosen-Ansorena D, Mifsud B, Pazoki R, Gao H, Ntritsos G, Dimou N, Cabrera CP, Karaman I, Ng FL, Evangelou M, Witkowska K, Tzanis E, Hellwege JN, Giri A, Velez Edwards DR, Sun YV, Cho K, Gaziano JM, Wilson PWF, Tsao PS, Kovesdy CP, Esko T, Mägi R, Milani L, Almgren P, Boutin T, Debette S, Ding J, Giulianini F, Holliday EG, Jackson AU, Li-Gao R, Lin WY, Luan J’, Mangino M, Oldmeadow C, Prins BP, Qian Y, Sargurupremraj M, Shah N, Surendran P, Thériault S, Verweij N, Willems SM, Zhao JH, Amouyel P, Connell J, de Mutsert R, Doney ASF, Farrall M, Menni C, Morris AD, Noordam R, Paré G, Poulter NR, Shields DC, Stanton A, Thom S, Abecasis G, Amin N, Arking DE, Ayers KL, Barbieri CM, Batini C, Bis JC, Blake T, Bochud M, Boehnke M, Boerwinkle E, Boomsma DI, Bottinger EP, Braund PS, Brumat M, Campbell A, Campbell H, Chakravarti A, Chambers JC, Chauhan G, Ciullo M, Cocca M, Collins F, Cordell HJ, Davies G, de Borst MH, de Geus EJ, Deary IJ, Deelen J, del Greco M. F, Demirkale CY, Dörr M, Ehret GB, Elosua R, Enroth S, Erzurumluoglu AM, Ferreira T, Frånberg M, Franco OH, Gandin I, Gasparini P, Giedraitis V, Gieger C, Girotto G, Goel A, Gow AJ, Gudnason V, Guo X, Gyllensten U, Hamsten A, Harris TB, Harris SE, Hartman CA, Havulinna AS, Hicks AA, Hofer E, Hofman A, Hottenga JJ, Huffman JE, Hwang SJ, Ingelsson E, James A, Jansen R, Jarvelin MR, Joehanes R, Johansson Å, Johnson AD, Joshi PK, Jousilahti P, Jukema JW, Jula A, Kähönen M, Kathiresan S, Keavney BD, Khaw KT, Knekt P, Knight J, Kolcic I, Kooner JS, Koskinen S, Kristiansson K, Kutalik Z, Laan M, Larson M, Launer LJ, Lehne B, Lehtimäki T, Liewald DCM, Lin L, Lind L, Lindgren CM, Liu YM, Loos RJF, Lopez LM, Lu Y, Lyytikäinen LP, Mahajan A, Mamasoula C, Marrugat J, Marten J, Milaneschi Y, Morgan A, Morris AP, Morrison AC, Munson PJ, Nalls MA, Nandakumar P, Nelson CP, Niiranen T, Nolte IM, Nutile T, Oldehinkel AJ, Oostra BA, O’Reilly PF, Org E, Padmanabhan S, Palmas W, Palotie A, Pattie A, Penninx BWJH, Perola M, Peters A, Polasek O, Pramstaller PP, Nguyen QT, Raitakari OT, Ren M, Rettig R, Rice K, Ridker PM, Ried JS, Riese H, Ripatti S, Robino A, Rose LM, Rotter JI, Rudan I, Ruggiero D, Saba Y, Sala CF, Salomaa V, Samani NJ, Sarin AP, Schmidt R, Schmidt H, Shrine N, Siscovick D, Smith AV, Snieder H, Sõber S, Sorice R, Starr JM, Stott DJ, Strachan DP, Strawbridge RJ, Sundström J, Swertz MA, Taylor KD, Teumer A, Tobin MD, Tomaszewski M, Toniolo D, Traglia M, Trompet S, Tuomilehto J, Tzourio C, Uitterlinden AG, Vaez A, van der Most PJ, van Duijn CM, Vergnaud AC, Verwoert GC, Vitart V, Völker U, Vollenweider P, Vuckovic D, Watkins H, Wild SH, Willemsen G, Wilson JF, Wright AF, Yao J, Zemunik T, Zhang W, Attia JR, Butterworth AS, Chasman DI, Conen D, Cucca F, Danesh J, Hayward C, Howson JMM, Laakso M, Lakatta EG, Langenberg C, Melander O, Mook-Kanamori DO, Palmer CNA, Risch L, Scott RA, Scott RJ, Sever P, Spector TD, van der Harst P, Wareham NJ, Zeggini E, Levy D, Munroe PB, Newton-Cheh C, Brown MJ, Metspalu A, Hung AM, O’Donnell CJ, Edwards TL, Psaty BM, Tzoulaki I, Barnes MR, Wain LV, Elliott P and Caulfield MJ (2018) Genetic analysis of over 1 million people identifies 535 new loci associated with blood pressure traits. Nature Genetics 50(10), 1412–1425. 30224653 10.1038/s41588-018-0205-x PMC6284793

[r131] Tindall AM, McLimans CJ, Petersen KS, Kris-Etherton PM and Lamendella R (2020) Walnuts and vegetable oils containing oleic acid differentially affect the gut microbiota and associations with cardiovascular risk factors: Follow-up of a randomized, controlled, feeding trial in adults at risk for cardiovascular disease. The Journal of Nutrition 150(4), 806–817. 31848609 10.1093/jn/nxz289 PMC7138683

[r132] Toral M, Robles-Vera I, de la Visitación N, Romero M, Sánchez M, Gómez-Guzmán M, Rodriguez-Nogales A, Yang T, Jiménez R, Algieri F, Gálvez J, Raizada MK and Duarte J (2019b) Role of the immune system in vascular function and blood pressure control induced by faecal microbiota transplantation in rats. Acta Physiologica 227(1), e13285. 31004464 10.1111/apha.13285

[r133] Toral M, Robles-Vera I, de la Visitación N, Romero M, Yang T, Sánchez M, Gómez-Guzmán M, Jiménez R, Raizada MK and Duarte J (2019a) Critical role of the interaction gut microbiota–sympathetic nervous system in the regulation of blood pressure. Frontiers in Physiology 10, 231. 30930793 10.3389/fphys.2019.00231 PMC6423906

[r134] Toral M, Romero M, Rodríguez-Nogales A, Jiménez R, Robles-Vera I, Algieri F, Chueca-Porcuna N, Sánchez M, de la Visitación N, Olivares M, García F, Pérez-Vizcaíno F, Gálvez J and Duarte J (2018) Lactobacillus fermentum improves tacrolimus‐induced hypertension by restoring vascular redox state and improving eNOS coupling. Molecular Nutrition & Food Research 62(14), 1800033. 10.1002/mnfr.201800033 29851248

[r135] Torkamani A, Wineinger NE and Topol EJ (2018) The personal and clinical utility of polygenic risk scores. Nature Reviews Genetics 19(9), 581–590. 10.1038/s41576-018-0018-x 29789686

[r136] Tsoi K, Yiu K, Lee H, Cheng HM, Wang TD, Tay JC, Teo BW, Turana Y, Soenarta AA, Sogunuru GP, Siddique S, Chia YC, Shin J, Chen CH, Wang JG, Kario K and the HOPE Asia Network (2021) Applications of artificial intelligence for hypertension management. The Journal of Clinical Hypertension 23(3), 568–574. 33533536 10.1111/jch.14180 PMC8029548

[r137] Understanding Society Scientific Group, International Consortium for Blood Pressure, Blood Pressure-International Consortium of Exome Chip Studies, Million Veteran Program, Giri A, Hellwege JN, Keaton JM, Park J, Qiu C, Warren HR, Torstenson ES, Kovesdy CP, Sun YV, Wilson OD, Robinson-Cohen C, Roumie CL, Chung CP, Birdwell KA, Damrauer SM, DuVall SL, Klarin D, Cho K, Wang Y, Evangelou E, Cabrera CP, Wain LV, Shrestha R, Mautz BS, Akwo EA, Sargurupremraj M, Debette S, Boehnke M, Scott LJ, Luan J’, Zhao JH, Willems SM, Thériault S, Shah N, Oldmeadow C, Almgren P, Li-Gao R, Verweij N, Boutin TS, Mangino M, Ntalla I, Feofanova E, Surendran P, Cook JP, Karthikeyan S, Lahrouchi N, Liu C, Sepúlveda N, Richardson TG, Kraja A, Amouyel P, Farrall M, Poulter NR, Laakso M, Zeggini E, Sever P, Scott RA, Langenberg C, Wareham NJ, Conen D, Palmer CNA, Attia J, Chasman DI, Ridker PM, Melander O, Mook-Kanamori DO, Harst P, Cucca F, Schlessinger D, Hayward C, Spector TD, Jarvelin MR, Hennig BJ, Timpson NJ, Wei WQ, Smith JC, Xu Y, Matheny ME, Siew EE, Lindgren C, Herzig KH, Dedoussis G, Denny JC, Psaty BM, Howson JMM, Munroe PB, Newton-Cheh C, Caulfield MJ, Elliott P, Gaziano JM, Concato J, Wilson PWF, Tsao PS, Velez Edwards DR, Susztak K, O’Donnell CJ, Hung AM and Edwards TL (2019) Trans-ethnic association study of blood pressure determinants in over 750,000 individuals. Nature Genetics 51(1), 51–62. 30578418 10.1038/s41588-018-0303-9 PMC6365102

[r138] Vaura F, Kauko A, Suvila K, Havulinna AS, Mars N, Salomaa V, FinnGen, Cheng S and Niiranen T (2021) Polygenic risk scores predict hypertension onset and cardiovascular risk. Hypertension 77(4), 1119–1127. 33611940 10.1161/HYPERTENSIONAHA.120.16471 PMC8025831

[r139] Verhaar BJH, Collard D, Prodan A, Levels JHM, Zwinderman AH, Bäckhed F, Vogt L, Peters MJL, Muller M, Nieuwdorp M and van den Born BJH (2020) Associations between gut microbiota, faecal short-chain fatty acids, and blood pressure across ethnic groups: The HELIUS study. European Heart Journal 41(44), 4259–4267. 32869053 10.1093/eurheartj/ehaa704 PMC7724641

[r140] Vijay-Kumar M, Aitken JD, Carvalho FA, Cullender TC, Mwangi S, Srinivasan S, Sitaraman SV, Knight R, Ley RE and Gewirtz AT (2010) Metabolic syndrome and altered gut microbiota in mice lacking toll-like receptor 5. Science 328(5975), 228–231. 20203013 10.1126/science.1179721 PMC4714868

[r141] Vos T, Lim SS, Abbafati C, Abbas KM, Abbasi M, Abbasifard M, Abbasi-Kangevari M, Abbastabar H, Abd-Allah F, Abdelalim A, Abdollahi M, Abdollahpour I, Abolhassani H, Aboyans V, Abrams EM, Abreu LG, Abrigo MRM, Abu-Raddad LJ, Abushouk AI, Acebedo A, Ackerman IN, Adabi M, Adamu AA, Adebayo OM, Adekanmbi V, Adelson JD, Adetokunboh OO, Adham D, Afshari M, Afshin A, Agardh EE, Agarwal G, Agesa KM, Aghaali M, Aghamir SMK, Agrawal A, Ahmad T, Ahmadi A, Ahmadi M, Ahmadieh H, Ahmadpour E, Akalu TY, Akinyemi RO, Akinyemiju T, Akombi B, al-Aly Z, Alam K, Alam N, Alam S, Alam T, Alanzi TM, Albertson SB, Alcalde-Rabanal JE, Alema NM, Ali M, Ali S, Alicandro G, Alijanzadeh M, Alinia C, Alipour V, Aljunid SM, Alla F, Allebeck P, Almasi-Hashiani A, Alonso J, al-Raddadi RM, Altirkawi KA, Alvis-Guzman N, Alvis-Zakzuk NJ, Amini S, Amini-Rarani M, Aminorroaya A, Amiri F, Amit AML, Amugsi DA, Amul GGH, Anderlini D, Andrei CL, Andrei T, Anjomshoa M, Ansari F, Ansari I, Ansari-Moghaddam A, Antonio CAT, Antony CM, Antriyandarti E, Anvari D, Anwer R, Arabloo J, Arab-Zozani M, Aravkin AY, Ariani F, Ärnlöv J, Aryal KK, Arzani A, Asadi-Aliabadi M, Asadi-Pooya AA, Asghari B, Ashbaugh C, Atnafu DD, Atre SR, Ausloos F, Ausloos M, Ayala Quintanilla BP, Ayano G, Ayanore MA, Aynalem YA, Azari S, Azarian G, Azene ZN, Babaee E, Badawi A, Bagherzadeh M, Bakhshaei MH, Bakhtiari A, Balakrishnan S, Balalla S, Balassyano S, Banach M, Banik PC, Bannick MS, Bante AB, Baraki AG, Barboza MA, Barker-Collo SL, Barthelemy CM, Barua L, Barzegar A, Basu S, Baune BT, Bayati M, Bazmandegan G, Bedi N, Beghi E, Béjot Y, Bello AK, Bender RG, Bennett DA, Bennitt FB, Bensenor IM, Benziger CP, Berhe K, Bernabe E, Bertolacci GJ, Bhageerathy R, Bhala N, Bhandari D, Bhardwaj P, Bhattacharyya K, Bhutta ZA, Bibi S, Biehl MH, Bikbov B, Bin Sayeed MS, Biondi A, Birihane BM, Bisanzio D, Bisignano C, Biswas RK, Bohlouli S, Bohluli M, Bolla SRR, Boloor A, Boon-Dooley AS, Borges G, Borzì AM, Bourne R, Brady OJ, Brauer M, Brayne C, Breitborde NJK, Brenner H, Briant PS, Briggs AM, Briko NI, Britton GB, Bryazka D, Buchbinder R, Bumgarner BR, Busse R, Butt ZA, Caetano dos Santos FL, Cámera LLAA, Campos-Nonato IR, Car J, Cárdenas R, Carreras G, Carrero JJ, Carvalho F, Castaldelli-Maia JM, Castañeda-Orjuela CA, Castelpietra G, Castle CD, Castro F, Catalá-López F, Causey K, Cederroth CR, Cercy KM, Cerin E, Chandan JS, Chang AR, Charlson FJ, Chattu VK, Chaturvedi S, Chimed-Ochir O, Chin KL, Cho DY, Christensen H, Chu DT, Chung MT, Cicuttini FM, Ciobanu LG, Cirillo M, Collins EL, Compton K, Conti S, Cortesi PA, Costa VM, Cousin E, Cowden RG, Cowie BC, Cromwell EA, Cross DH, Crowe CS, Cruz JA, Cunningham M, Dahlawi SMA, Damiani G, Dandona L, Dandona R, Darwesh AM, Daryani A, das JK, das Gupta R, das Neves J, Dávila-Cervantes CA, Davletov K, de Leo D, Dean FE, DeCleene NK, Deen A, Degenhardt L, Dellavalle RP, Demeke FM, Demsie DG, Denova-Gutiérrez E, Dereje ND, Dervenis N, Desai R, Desalew A, Dessie GA, Dharmaratne SD, Dhungana GP, Dianatinasab M, Diaz D, Dibaji Forooshani ZS, Dingels ZV, Dirac MA, Djalalinia S, do HT, Dokova K, Dorostkar F, Doshi CP, Doshmangir L, Douiri A, Doxey MC, Driscoll TR, Dunachie SJ, Duncan BB, Duraes AR, Eagan AW, Ebrahimi Kalan M, Edvardsson D, Ehrlich JR, el Nahas N, el Sayed I, el Tantawi M, Elbarazi I, Elgendy IY, Elhabashy HR, el-Jaafary SI, Elyazar IRF, Emamian MH, Emmons-Bell S, Erskine HE, Eshrati B, Eskandarieh S, Esmaeilnejad S, Esmaeilzadeh F, Esteghamati A, Estep K, Etemadi A, Etisso AE, Farahmand M, Faraj A, Fareed M, Faridnia R, Farinha CSS, Farioli A, Faro A, Faruque M, Farzadfar F, Fattahi N, Fazlzadeh M, Feigin VL, Feldman R, Fereshtehnejad SM, Fernandes E, Ferrari AJ, Ferreira ML, Filip I, Fischer F, Fisher JL, Fitzgerald R, Flohr C, Flor LS, Foigt NA, Folayan MO, Force LM, Fornari C, Foroutan M, Fox JT, Freitas M, Fu W, Fukumoto T, Furtado JM, Gad MM, Gakidou E, Galles NC, Gallus S, Gamkrelidze A, Garcia-Basteiro AL, Gardner WM, Geberemariyam BS, Gebrehiwot AM, Gebremedhin KB, Gebreslassie AAAA, Gershberg Hayoon A, Gething PW, Ghadimi M, Ghadiri K, Ghafourifard M, Ghajar A, Ghamari F, Ghashghaee A, Ghiasvand H, Ghith N, Gholamian A, Gilani SA, Gill PS, Gitimoghaddam M, Giussani G, Goli S, Gomez RS, Gopalani SV, Gorini G, Gorman TM, Gottlich HC, Goudarzi H, Goulart AC, Goulart BNG, Grada A, Grivna M, Grosso G, Gubari MIM, Gugnani HC, Guimaraes ALS, Guimarães RA, Guled RA, Guo G, Guo Y, Gupta R, Haagsma JA, Haddock B, Hafezi-Nejad N, Hafiz A, Hagins H, Haile LM, Hall BJ, Halvaei I, Hamadeh RR, Hamagharib Abdullah K, Hamilton EB, Han C, Han H, Hankey GJ, Haro JM, Harvey JD, Hasaballah AI, Hasanzadeh A, Hashemian M, Hassanipour S, Hassankhani H, Havmoeller RJ, Hay RJ, Hay SI, Hayat K, Heidari B, Heidari G, Heidari-Soureshjani R, Hendrie D, Henrikson HJ, Henry NJ, Herteliu C, Heydarpour F, Hird TR, Hoek HW, Hole MK, Holla R, Hoogar P, Hosgood HD, Hosseinzadeh M, Hostiuc M, Hostiuc S, Househ M, Hoy DG, Hsairi M, Hsieh VCR, Hu G, Huda TM, Hugo FN, Huynh CK, Hwang BF, Iannucci VC, Ibitoye SE, Ikuta KS, Ilesanmi OS, Ilic IM, Ilic MD, Inbaraj LR, Ippolito H, Irvani SSN, Islam MM, Islam MM, Islam SMS, Islami F, Iso H, Ivers RQ, Iwu CCD, Iyamu IO, Jaafari J, Jacobsen KH, Jadidi-Niaragh F, Jafari H, Jafarinia M, Jahagirdar D, Jahani MA, Jahanmehr N, Jakovljevic M, Jalali A, Jalilian F, James SL, Janjani H, Janodia MD, Jayatilleke AU, Jeemon P, Jenabi E, Jha RP, Jha V, Ji JS, Jia P, John O, John-Akinola YO, Johnson CO, Johnson SC, Jonas JB, Joo T, Joshi A, Jozwiak JJ, Jürisson M, Kabir A, Kabir Z, Kalani H, Kalani R, Kalankesh LR, Kalhor R, Kamiab Z, Kanchan T, Karami Matin B, Karch A, Karim MA, Karimi SE, Kassa GM, Kassebaum NJ, Katikireddi SV, Kawakami N, Kayode GA, Keddie SH, Keller C, Kereselidze M, Khafaie MA, Khalid N, Khan M, Khatab K, Khater MM, Khatib MN, Khayamzadeh M, Khodayari MT, Khundkar R, Kianipour N, Kieling C, Kim D, Kim YE, Kim YJ, Kimokoti RW, Kisa A, Kisa S, Kissimova-Skarbek K, Kivimäki M, Kneib CJ, Knudsen AKS, Kocarnik JM, Kolola T, Kopec JA, Kosen S, Koul PA, Koyanagi A, Kravchenko MA, Krishan K, Krohn KJ, Kuate Defo B, Kucuk Bicer B, Kumar GA, Kumar M, Kumar P, Kumar V, Kumaresh G, Kurmi OP, Kusuma D, Kyu HH, la Vecchia C, Lacey B, Lal DK, Lalloo R, Lam JO, Lami FH, Landires I, Lang JJ, Lansingh VC, Larson SL, Larsson AO, Lasrado S, Lassi ZS, Lau KMM, Lavados PM, Lazarus JV, Ledesma JR, Lee PH, Lee SWH, LeGrand KE, Leigh J, Leonardi M, Lescinsky H, Leung J, Levi M, Lewington S, Li S, Lim LL, Lin C, Lin RT, Linehan C, Linn S, Liu HC, Liu S, Liu Z, Looker KJ, Lopez AD, Lopukhov PD, Lorkowski S, Lotufo PA, Lucas TCD, Lugo A, Lunevicius R, Lyons RA, Ma J, MacLachlan JH, Maddison ER, Maddison R, Madotto F, Mahasha PW, Mai HT, Majeed A, Maled V, Maleki S, Malekzadeh R, Malta DC, Mamun AA, Manafi A, Manafi N, Manguerra H, Mansouri B, Mansournia MA, Mantilla Herrera AM, Maravilla JC, Marks A, Martins-Melo FR, Martopullo I, Masoumi SZ, Massano J, Massenburg BB, Mathur MR, Maulik PK, McAlinden C, McGrath JJ, McKee M, Mehndiratta MM, Mehri F, Mehta KM, Meitei WB, Memiah PTN, Mendoza W, Menezes RG, Mengesha EW, Mengesha MB, Mereke A, Meretoja A, Meretoja TJ, Mestrovic T, Miazgowski B, Miazgowski T, Michalek IM, Mihretie KM, Miller TR, Mills EJ, Mirica A, Mirrakhimov EM, Mirzaei H, Mirzaei M, Mirzaei-Alavijeh M, Misganaw AT, Mithra P, Moazen B, Moghadaszadeh M, Mohamadi E, Mohammad DK, Mohammad Y, Mohammad Gholi Mezerji N, Mohammadian-Hafshejani A, Mohammadifard N, Mohammadpourhodki R, Mohammed S, Mokdad AH, Molokhia M, Momen NC, Monasta L, Mondello S, Mooney MD, Moosazadeh M, Moradi G, Moradi M, Moradi-Lakeh M, Moradzadeh R, Moraga P, Morales L, Morawska L, Moreno Velásquez I, Morgado-da-Costa J, Morrison SD, Mosser JF, Mouodi S, Mousavi SM, Mousavi Khaneghah A, Mueller UO, Munro SB, Muriithi MK, Musa KI, Muthupandian S, Naderi M, Nagarajan AJ, Nagel G, Naghshtabrizi B, Nair S, Nandi AK, Nangia V, Nansseu JR, Nayak VC, Nazari J, Negoi I, Negoi RI, Netsere HBN, Ngunjiri JW, Nguyen CT, Nguyen J, Nguyen M, Nguyen M, Nichols E, Nigatu D, Nigatu YT, Nikbakhsh R, Nixon MR, Nnaji CA, Nomura S, Norrving B, Noubiap JJ, Nowak C, Nunez-Samudio V, Oţoiu A, Oancea B, Odell CM, Ogbo FA, Oh IH, Okunga EW, Oladnabi M, Olagunju AT, Olusanya BO, Olusanya JO, Oluwasanu MM, Omar Bali A, Omer MO, Ong KL, Onwujekwe OE, Orji AU, Orpana HM, Ortiz A, Ostroff SM, Otstavnov N, Otstavnov SS, Øverland S, Owolabi MO, P A M, Padubidri JR, Pakhare AP, Palladino R, Pana A, Panda-Jonas S, Pandey A, Park EK, Parmar PGK, Pasupula DK, Patel SK, Paternina-Caicedo AJ, Pathak A, Pathak M, Patten SB, Patton GC, Paudel D, Pazoki Toroudi H, Peden AE, Pennini A, Pepito VCF, Peprah EK, Pereira A, Pereira DM, Perico N, Pham HQ, Phillips MR, Pigott DM, Pilgrim T, Pilz TM, Pirsaheb M, Plana-Ripoll O, Plass D, Pokhrel KN, Polibin RV, Polinder S, Polkinghorne KR, Postma MJ, Pourjafar H, Pourmalek F, Pourmirza Kalhori R, Pourshams A, Poznańska A, Prada SI, Prakash V, Pribadi DRA, Pupillo E, Quazi Syed Z, Rabiee M, Rabiee N, Radfar A, Rafiee A, Rafiei A, Raggi A, Rahimi-Movaghar A, Rahman MA, Rajabpour-Sanati A, Rajati F, Ramezanzadeh K, Ranabhat CL, Rao PC, Rao SJ, Rasella D, Rastogi P, Rathi P, Rawaf DL, Rawaf S, Rawal L, Razo C, Redford SB, Reiner Jr RC, Reinig N, Reitsma MB, Remuzzi G, Renjith V, Renzaho AMN, Resnikoff S, Rezaei N, Rezai M, Rezapour A, Rhinehart PA, Riahi SM, Ribeiro ALP, Ribeiro DC, Ribeiro D, Rickard J, Roberts NLS, Roberts S, Robinson SR, Roever L, Rolfe S, Ronfani L, Roshandel G, Roth GA, Rubagotti E, Rumisha SF, Sabour S, Sachdev PS, Saddik B, Sadeghi E, Sadeghi M, Saeidi S, Safi S, Safiri S, Sagar R, Sahebkar A, Sahraian MA, Sajadi SM, Salahshoor MR, Salamati P, Salehi Zahabi S, Salem H, Salem MRR, Salimzadeh H, Salomon JA, Salz I, Samad Z, Samy AM, Sanabria J, Santomauro DF, Santos IS, Santos JV, Santric-Milicevic MM, Saraswathy SYI, Sarmiento-Suárez R, Sarrafzadegan N, Sartorius B, Sarveazad A, Sathian B, Sathish T, Sattin D, Sbarra AN, Schaeffer LE, Schiavolin S, Schmidt MI, Schutte AE, Schwebel DC, Schwendicke F, Senbeta AM, Senthilkumaran S, Sepanlou SG, Shackelford KA, Shadid J, Shahabi S, Shaheen AA, Shaikh MA, Shalash AS, Shams-Beyranvand M, Shamsizadeh M, Shannawaz M, Sharafi K, Sharara F, Sheena BS, Sheikhtaheri A, Shetty RS, Shibuya K, Shiferaw WS, Shigematsu M, Shin JI, Shiri R, Shirkoohi R, Shrime MG, Shuval K, Siabani S, Sigfusdottir ID, Sigurvinsdottir R, Silva JP, Simpson KE, Singh A, Singh JA, Skiadaresi E, Skou ST, Skryabin VY, Sobngwi E, Sokhan A, Soltani S, Sorensen RJD, Soriano JB, Sorrie MB, Soyiri IN, Sreeramareddy CT, Stanaway JD, Stark BA, Ştefan SC, Stein C, Steiner C, Steiner TJ, Stokes MA, Stovner LJ, Stubbs JL, Sudaryanto A, Sufiyan M’B, Sulo G, Sultan I, Sykes BL, Sylte DO, Szócska M, Tabarés-Seisdedos R, Tabb KM, Tadakamadla SK, Taherkhani A, Tajdini M, Takahashi K, Taveira N, Teagle WL, Teame H, Tehrani-Banihashemi A, Teklehaimanot BF, Terrason S, Tessema ZT, Thankappan KR, Thomson AM, Tohidinik HR, Tonelli M, Topor-Madry R, Torre AE, Touvier M, Tovani-Palone MRR, Tran BX, Travillian R, Troeger CE, Truelsen TC, Tsai AC, Tsatsakis A, Tudor Car L, Tyrovolas S, Uddin R, Ullah S, Undurraga EA, Unnikrishnan B, Vacante M, Vakilian A, Valdez PR, Varughese S, Vasankari TJ, Vasseghian Y, Venketasubramanian N, Violante FS, Vlassov V, Vollset SE, Vongpradith A, Vukovic A, Vukovic R, Waheed Y, Walters MK, Wang J, Wang Y, Wang YP, Ward JL, Watson A, Wei J, Weintraub RG, Weiss DJ, Weiss J, Westerman R, Whisnant JL, Whiteford HA, Wiangkham T, Wiens KE, Wijeratne T, Wilner LB, Wilson S, Wojtyniak B, Wolfe CDA, Wool EE, Wu AM, Wulf Hanson S, Wunrow HY, Xu G, Xu R, Yadgir S, Yahyazadeh Jabbari SH, Yamagishi K, Yaminfirooz M, Yano Y, Yaya S, Yazdi-Feyzabadi V, Yearwood JA, Yeheyis TY, Yeshitila YG, Yip P, Yonemoto N, Yoon SJ, Yoosefi Lebni J, Younis MZ, Younker TP, Yousefi Z, Yousefifard M, Yousefinezhadi T, Yousuf AY, Yu C, Yusefzadeh H, Zahirian Moghadam T, Zaki L, Zaman SB, Zamani M, Zamanian M, Zandian H, Zangeneh A, Zastrozhin MS, Zewdie KA, Zhang Y, Zhang ZJ, Zhao JT, Zhao Y, Zheng P, Zhou M, Ziapour A, Zimsen SRM, Naghavi M and Murray CJL (2020) Global burden of 369 diseases and injuries in 204 countries and territories, 1990–2019: A systematic analysis for the global burden of disease study 2019. The Lancet 396(10258), 1204–1222. 10.1016/S0140-6736(20)30925-9 PMC7567026 33069326

[r142] Waghulde H, Cheng X, Galla S, Mell B, Cai J, Pruett-Miller SM, Vazquez G, Patterson A, Vijay Kumar M and Joe B (2018) Attenuation of microbiotal dysbiosis and hypertension in a CRISPR/Cas9 gene ablation rat model of GPER1. Hypertension 72(5), 1125–1132. 30354811 10.1161/HYPERTENSIONAHA.118.11175 PMC6208154

[r143] Walejko JM, Kim S, Goel R, Handberg EM, Richards EM, Pepine CJ and Raizada MK (2018) Gut microbiota and serum metabolite differences in African Americans and white Americans with high blood pressure. International Journal of Cardiology 271, 336–339. 30049487 10.1016/j.ijcard.2018.04.074 PMC6143419

[r144] Wan Y, Jiang J, Lu M, Tong W, Zhou R, Li J, Yuan J, Wang F and Li D (2020) Human milk microbiota development during lactation and its relation to maternal geographic location and gestational hypertensive status. Gut Microbes 11(5), 1438–1449. 32543266 10.1080/19490976.2020.1760711 PMC7524296

[r145] Wan C, Zhu C, Jin G, Zhu M, Hua J and He Y (2021) Analysis of gut microbiota in patients with coronary artery disease and hypertension. Evidence-based Complementary and Alternative Medicine 2021, 7195082. 34987598 10.1155/2021/7195082 PMC8723847

[r146] Wang B, Liu J, Lei R, Xue B, Li Y, Tian X, Zhang K and Luo B (2022) Cold exposure, gut microbiota, and hypertension: A mechanistic study. Science of the Total Environment 833, 155199. 35417730 10.1016/j.scitotenv.2022.155199

[r147] Wang Z, Tang WHW, Buffa JA, Fu X, Britt EB, Koeth RA, Levison BS, Fan Y, Wu Y and Hazen SL (2014) Prognostic value of choline and betaine depends on intestinal microbiota-generated metabolite trimethylamine-*N*-oxide. European Heart Journal 35(14), 904–910. 24497336 10.1093/eurheartj/ehu002 PMC3977137

[r148] Wang J, Yang M, Wu Q, Chen J, Deng SF, Chen L, Wei DN and Liang F (2021) Improvement of intestinal flora: Accompany with the antihypertensive effect of electroacupuncture on stage 1 hypertension. Chinese Medicine 16(1), 1–11. 33413552 10.1186/s13020-020-00417-8 PMC7792359

[r149] Warren HR, Evangelou E, Cabrera CP, Gao H, Ren M, Mifsud B, Ntalla I, Surendran P, Liu C, Cook JP, Kraja AT, Drenos F, Loh M, Verweij N, Marten J, Karaman I, Lepe MP, O’Reilly PF, Knight J, Snieder H, Kato N, He J, Tai ES, Said MA, Porteous D, Alver M, Poulter N, Farrall M, Gansevoort RT, Padmanabhan S, Mägi R, Stanton A, Connell J, Bakker SJ, Metspalu A, Shields DC, Thom S, Brown M, Sever P, Esko T, Hayward C, van der Harst P, Saleheen D, Chowdhury R, Chambers JC, Chasman DI, Chakravarti A, Newton-Cheh C, Lindgren CM, Levy D, Kooner JS, Keavney B, Tomaszewski M, Samani NJ, Howson JM, Tobin MD, Munroe PB, Ehret GB, Wain LV, International Consortium of Blood Pressure (ICBP) 1000G Analyses, BIOS Consortium, Lifelines Cohort Study, Understanding Society Scientific group, CHD Exome+ Consortium, ExomeBP Consortium, T2D-GENES Consortium, GoT2DGenes Consortium, Cohorts for Heart and Ageing Research in Genome Epidemiology (CHARGE) BP Exome Consortium, International Genomics of Blood Pressure (iGEN-BP) Consortium and UK Biobank CardioMetabolic Consortium BP working group (2017) Genome-wide association analysis identifies novel blood pressure loci and offers biological insights into cardiovascular risk. Nature Genetics 49(3), 403–415. 28135244 10.1038/ng.3768 PMC5972004

[r150] Wellcome Trust Case Control Consortium (2007) Genome-wide association study of 14,000 cases of seven common diseases and 3,000 shared controls. Nature 447(7145), 661–678. 17554300 10.1038/nature05911 PMC2719288

[r151] Weng Z, Liu Q, Yan Q, Liang J, Zhang X, Xu J, Li W, Xu C and Gu A (2022) Associations of genetic risk factors and air pollution with incident hypertension among participants in the UK biobank study. Chemosphere 299, 134398. 35339527 10.1016/j.chemosphere.2022.134398

[r152] Wilck N, Matus MG, Kearney SM, Olesen SW, Forslund K, Bartolomaeus H, Haase S, Mähler A, Balogh A, Markó L, Vvedenskaya O, Kleiner FH, Tsvetkov D, Klug L, Costea PI, Sunagawa S, Maier L, Rakova N, Schatz V, Neubert P, Frätzer C, Krannich A, Gollasch M, Grohme DA, Côrte-Real BF, Gerlach RG, Basic M, Typas A, Wu C, Titze JM, Jantsch J, Boschmann M, Dechend R, Kleinewietfeld M, Kempa S, Bork P, Linker RA, Alm EJ and Müller DN (2017) Salt-responsive gut commensal modulates TH17 axis and disease. Nature 551(7682), 585–589. 29143823 10.1038/nature24628 PMC6070150

[r153] Wu H, Lam TYC, Shum T-F, Tsai T-Y and Chiou J (2022) Hypotensive effect of captopril on deoxycorticosterone acetate-salt-induced hypertensive rat is associated with gut microbiota alteration. Hypertension Research 45(2), 270–282. 34857899 10.1038/s41440-021-00796-x PMC8766282

[r154] Xia W-J, Xu M-L, Yu X-J, du MM, Li XH, Yang T, Li L, Li Y, Kang KB, Su Q, Xu JX, Shi XL, Wang XM, Li HB and Kang YM (2021) Antihypertensive effects of exercise involve reshaping of gut microbiota and improvement of gut-brain axis in spontaneously hypertensive rat. Gut Microbes 13(1), 1–24. 10.1080/19490976.2020.1854642 PMC7781639 33382364

[r155] Xu C and Marques FZ (2022) How dietary fibre, acting via the gut microbiome, lowers blood pressure. Current Hypertension Reports 24, 509–521. 35838884 10.1007/s11906-022-01216-2 PMC9568477

[r156] Yan Q, Gu Y, Li X, Yang W, Jia L, Chen C, Han X, Huang Y, Zhao L, Li P, Fang Z, Zhou J, Guan X, Ding Y, Wang S, Khan M, Xin Y, Li S and Ma Y (2017) Alterations of the gut microbiome in hypertension. Frontiers in Cellular and Infection Microbiology 7, 381. 28884091 10.3389/fcimb.2017.00381 PMC5573791

[r157] Yan X, Jin J, Su X, Yin X, Gao J, Wang X, Zhang S, Bu P, Wang M, Zhang Y, Wang Z and Zhang Y (2020) Intestinal flora modulates blood pressure by regulating the synthesis of intestinal-derived corticosterone in high salt-induced hypertension. Circulation Research 126(7), 839–853. 32078445 10.1161/CIRCRESAHA.119.316394

[r158] Yang T, Ahmari N, Schmidt JT, Redler T, Arocha R, Pacholec K, Magee KL, Malphurs W, Owen JL, Krane GA, Li E, Wang GP, Vickroy TW, Raizada MK, Martyniuk CJ and Zubcevic J (2017) Shifts in the gut microbiota composition due to depleted bone marrow beta adrenergic signaling are associated with suppressed inflammatory transcriptional networks in the mouse colon. Frontiers in Physiology 8, 220. 28446880 10.3389/fphys.2017.00220 PMC5388758

[r159] Yang T, Aquino V, Lobaton GO, Li H, Colon-Perez L, Goel R, Qi Y, Zubcevic J, Febo M, Richards EM, Pepine CJ and Raizada MK (2019a) Sustained captopril‐induced reduction in blood pressure is associated with alterations in gut‐brain axis in the spontaneously hypertensive rat. Journal of the American Heart Association 8(4), e010721. 30755073 10.1161/JAHA.118.010721 PMC6405665

[r160] Yang T, Li H, Oliveira AC, Goel R, Richards EM, Pepine CJ and Raizada MK (2020) Transcriptomic signature of gut microbiome-contacting cells in colon of spontaneously hypertensive rats. Physiological Genomics 52(3), 121–132. 31869283 10.1152/physiolgenomics.00087.2019 PMC7099409

[r161] Yang T, Magee KL, Colon-Perez LM, Larkin R, Liao YS, Balazic E, Cowart JR, Arocha R, Redler T, Febo M, Vickroy T, Martyniuk CJ, Reznikov LR and Zubcevic J (2019b) Impaired butyrate absorption in the proximal colon, low serum butyrate and diminished central effects of butyrate on blood pressure in spontaneously hypertensive rats. Acta Physiologica 226(2), e13256. 30656835 10.1111/apha.13256 PMC7199780

[r162] Yang T, Mei X, Tackie-Yarboi E, Akere MT, Kyoung J, Mell B, Yeo JY, Cheng X, Zubcevic J, Richards EM, Pepine CJ, Raizada MK, Schiefer IT and Joe B (2022) Identification of a gut commensal that compromises the blood pressure-lowering effect of Ester angiotensin-converting enzyme inhibitors. Hypertension 79, 1591–1601 35538603 10.1161/HYPERTENSIONAHA.121.18711 PMC9278702

[r163] Yang T, Richards EM, Pepine CJ and Raizada MK (2018) The gut microbiota and the brain–gut–kidney axis in hypertension and chronic kidney disease. Nature Reviews Nephrology 14(7), 442–456. 29760448 10.1038/s41581-018-0018-2 PMC6385605

[r164] Yang T, Santisteban MM, Rodriguez V, Li E, Ahmari N, Carvajal JM, Zadeh M, Gong M, Qi Y, Zubcevic J, Sahay B, Pepine CJ, Raizada MK and Mohamadzadeh M (2015) Gut dysbiosis is linked to hypertension. Hypertension 65(6), 1331–1340. 25870193 10.1161/HYPERTENSIONAHA.115.05315 PMC4433416

[r165] Ye C, Fu T, Hao S, Zhang Y, Wang O, Jin B, Xia M, Liu M, Zhou X, Wu Q, Guo Y, Zhu C, Li YM, Culver DS, Alfreds ST, Stearns F, Sylvester KG, Widen E, McElhinney D and Ling X (2018) Prediction of incident hypertension within the next year: Prospective study using statewide electronic health records and machine learning. Journal of Medical Internet Research 20(1), e9268. 10.2196/jmir.9268 PMC5811646 29382633

[r166] Zeng X, Xing X, Gupta M, Keber FC, Lopez JG, Lee YCJ, Roichman A, Wang L, Neinast MD, Donia MS, Wühr M, Jang C and Rabinowitz JD (2022) Gut bacterial nutrient preferences quantified in vivo. Cell 185(18), 3441–3456. 36055202 10.1016/j.cell.2022.07.020 PMC9450212

[r167] Zheng T, Wu Y, Peng M, Xiao N, Tan Z and Yang T (2022) Hypertension of liver-yang hyperactivity syndrome induced by a high salt diet by altering components of the gut microbiota associated with the glutamate/GABA-glutamine cycle. Frontiers in Nutrition 9, 964273. 36017217 10.3389/fnut.2022.964273 PMC9395663

[r168] Zhong H-J, Zeng H-L, Cai Y-L, Zhuang YP, Liou YL, Wu Q and He X-X (2021) Washed microbiota transplantation lowers blood pressure in patients with hypertension. Frontiers in Cellular and Infection Microbiology 11, 679624. 34458158 10.3389/fcimb.2021.679624 PMC8385408

